# Alternative stable states in the intestinal ecosystem: proof of concept in a rat model and a perspective of therapeutic implications

**DOI:** 10.1186/s40168-020-00933-7

**Published:** 2020-11-06

**Authors:** Maarten Van de Guchte, Sebastian D. Burz, Julie Cadiou, Jiangbo Wu, Stanislas Mondot, Hervé M. Blottière, Joël Doré

**Affiliations:** 1 University Paris-Saclay, INRAE, AgroParisTech, Micalis Institute, 78350 Jouy-en-Josas, France; 2 University Paris-Saclay, INRAE, Metagenopolis, 78350 Jouy-en-Josas, France

**Keywords:** Alternative stable states, Intestinal ecosystem, GI tract, Microbiota, Host, Symbiosis, Interaction, Inflammation, Therapy, Cure

## Abstract

**Background:**

Chronic immune-mediated diseases are rapidly expanding and notoriously difficult to cure. Altered relatively stable intestinal microbiota configurations are associated with several of these diseases, and with a possible pre-disease condition (more susceptible to disease development) of the host-microbiota ecosystem. These observations are reminiscent of the behavior of an ecosystem with alternative stable states (different stable configurations that can exist under identical external conditions), and we recently postulated that health, pre-disease and disease represent such alternative states. Here, our aim was to examine if alternative stable states indeed exist in the intestinal ecosystem.

**Results:**

Rats were exposed to varying concentrations of DSS in order to create a wide range of mildly inflammatory conditions, in a context of diet-induced low microbiota diversity. The consequences for the intestinal microbiota were traced by 16S rRNA gene profiling over time, and inflammation of the distal colon was evaluated at sacrifice, 45 days after the last DSS treatment. The results provide the first formal experimental proof for the existence of alternative stable states in the rat intestinal ecosystem, taking both microbiota and host inflammatory status into consideration. The alternative states are host-microbiota ecosystem states rather than independent and dissociated microbiota and host states, and inflammation can prompt stable state-transition. Based on these results, we propose a conceptual model providing new insights in the interplay between host inflammatory status and microbiota status. These new insights call for innovative therapeutic strategies to cure (pre-)disease.

**Conclusions:**

We provide proof of concept showing the existence of alternative stable states in the rat intestinal ecosystem. We further propose a model which, if validated in humans, will support innovative diagnosis, therapeutic strategy, and monitoring in the treatment of chronic inflammatory conditions. This model provides a strong rationale for the application of combinatorial therapeutic strategies, targeting host and microbiota rather than only one of the two in chronic immune-mediated diseases.

Video Abstract

**Supplementary information:**

**Supplementary information** accompanies this paper at 10.1186/s40168-020-00933-7.

## Background

The past decade has seen remarkable progress in our knowledge of the human intestinal ecosystem, revealing an intimate relationship between humans and their gut microbiota. Non-pathogenic commensal and transiting bacteria prove to influence fundamental host processes, including metabolism, adiposity, maturation and modulation of the immune system, and even brain function and decision-making [1–5]. The host employs a network of dedicated receptors and signaling pathways to capture information from the microbiota and the gut environment and respond in an appropriate way.

Intestinal microbiota assemblies are not entirely random or equally distributed. Configurations of relative microbial taxa abundances can be recognized that occur more frequently than others [6–9]. When human gut microbiota samples are classified by microbial “gene richness”, a measure of microbiota diversity, a bimodal distribution is observed with microbiota having either a low (LGC) or a high gene count (HGC) [10], where LGC individuals appear to be more prone to disease development or aggravation. More specific atypical microbiota compositions are associated with an ever-growing number of chronic inflammatory diseases. These may be characterized by (among other changes) bacterial species with particularly modified abundance (e.g., [11–13]), or by the alteration of a set of bacterial species that can together serve as a biomarker with high predictive value (e.g., [10]).

**Fig. 1.**
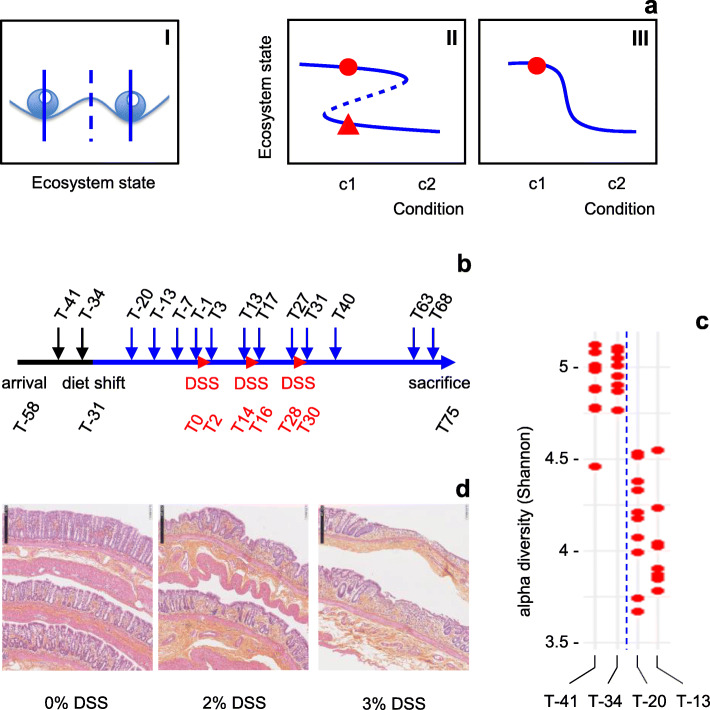
Experimental design. **a** Alternative stable states. Subpanel I, alternative stable states of an ecosystem as beads in a stability landscape. Dashed line, frontier between two basins of attraction. Subpanel II, alternative states (solid lines) can both exist under a range of identical conditions (bi-stable range). Dashed line, see subpanel I. Width and shape of the basins of attraction, and thereby the stability of the alternative states, change with changing conditions, as illustrated by the changing distances between solid lines and the dashed line [14]. When changing conditions push the ecosystem beyond a tipping point (sharp bend in the Z-shape curve), the limit of resilience where the basin of attraction of its present state disappears, it rapidly transits to an alternative state. Subpanel III, steep gradient where for any given condition only one stable state exists. Assuming that the original ecosystem state is represented by the red dot, models II and III both predict a change of ecosystem state when the external conditions change from c1 to c2. When the conditions change back to c1, model II predicts that the system remains in the alternative state (red triangle), while in model III the system returns to its original state. **b** Timeline of the experiment. Black and blue solid lines, different diets as indicated in the text (diet shift at T-31). Small arrows, fecal sampling time-points. DSS, DSS treatment periods (3 days each). T, time in days relative to start of first DSS treatment (T0). Reception of animals at T-58, sacrifice and distal colon histology at T75. **c** Reduction of microbiota diversity after diet shift (T-31). Time-points are indicated at the bottom of the figure. Each dot represents one rat. **d** Induction of low-grade inflammation through DSS treatments. Distal colon histology, 45 days after last DSS treatment. Size bar, 250 μm

Thus, a picture emerges of an intestinal ecosystem that can exist in several states, each characterized by specific microbiota and host parameters and representing health, pre-disease (more susceptible to develop disease), or different diseases. The relative constancy of these states is reminiscent of alternative stable states, different states that can exist under (a range of) identical external conditions (as opposed to different states that correspond to different external conditions, separated by a steep gradient (Fig. 1a)). As a consequence, while a shift to an alternative stable (pre-)disease state may be provoked when conditions change beyond the limits of system resilience, simply setting the conditions back to what they were before the transition does not revert system state to its initial configuration (if the initial conditions are within the bi-stable range, Fig. 1a) [14].

Western lifestyle (including diet and other factors) may have caused such a critical transition of the intestinal ecosystem to an alternative stable pre-disease state [15], thus creating permissive conditions for the marked, sometimes exponential, rise in the incidence of chronic immune-mediated diseases observed over the last 60 years [16]. If true, this hypothesis will have important implications, while at the same time opening conceptually new avenues, for prevention and therapy [15].

In the present study, we provide proof of concept for the existence of alternative stable states in a model intestinal ecosystem and show that intestinal barrier disrupting substances can induce critical transition in the context of a low-diversity microbiota. We propose a new theoretical framework to describe the interplay between host inflammatory status and microbiota status, both of which show bimodal distributions across our data and which together describe host-microbiota ecosystem status. According to our model, the latter can take the form of alternative stable states as described above, or of more fragile states of intermediate stability (lower resilience) which can be regarded as risk situations for health deterioration or, inversely, as therapeutic opportunities for health improvement.

## Results

### Diet shift leads to a marked change in gut microbiota diversity

We hypothesize that low microbiota diversity and inflammation due to intestinal barrier failure, both of which can be brought about by Western dietary habits or other risk factors, can push the ecosystem beyond a tipping point (Fig. 1a, subpanel II) and induce a self-enhancing process of alteration of the intestinal ecosystem, resulting in a transition to an alternative stable state (Additional Fig. 1) [15]. The present study examines if proof of concept can be obtained for the existence of alternative stable states and state-transition in the intestinal ecosystem.

For this purpose, we sought to create the abovementioned conditions of low microbiota diversity and inflammation in a rat model, while controlling external conditions. The latter aspect is essential to distinguish alternative stable states from different states resulting from different external conditions and separated by a steep gradient (Fig. 1a), but often hard to ascertain in descriptive studies of human populations, like those cited above which showed that intestinal microbiota assemblies are not random.

First, rats raised in individual cages and initially kept on a standard chow diet (Additional Table 1, diet 1) were adapted to a diet without crude fiber, with a slightly changed macronutrient composition (more sugar and fat, less protein; Additional Table 1, diet 2; Fig. 1b), in order to reduce the richness of the microbiota. An analysis of microbiota composition showed that this diet shift indeed produced the expected reduction of alpha diversity (Fig. 1c). The observed number of OTUs was reduced by about 25% (not shown). Further exploration indicated that the main effects of diet shift involved an increase in the relative abundance of the genera *Allobaculum*, *Desulfovibrio*, *Christensenella* and, to a lesser extent, *Bacteroides*, *Parabacteroides,* and *Butyricimonas*, and a decrease of the genera *Prevotella*, *Lactobacillus,* and *Saccharimonas* (Additional Fig. 2a; Additional Table 2). Within the genus *Lactobacillus*, abundance reductions were observed for *L. reuteri*, *L. murinus*, and *L. intestinalis* (Additional Fig. 2b).

### Transient ecosystem perturbation reveals two alternative microbiota states

In this context of diet-induced low microbiota diversity, the rats were exposed to three interspersed dextran sodium sulfate treatments (DSS, an intestinal barrier-disrupting compound commonly used to induce colitis in rodent models, in order to mimic ulcerative colitis in preclinical studies [17]) of 3 days each, over a period of 1 month (Fig. 1b: T0–T2, T14–T16, T28–T30). DSS was administered in drinking water, in concentrations ranging from 0 to 3% for different groups in order to create a wide range of mildly inflammatory conditions. This resulted for the highest concentration (3%) of DSS in a significant stagnation of bodyweight development during the first two treatment periods, which was recovered between treatments (Additional Fig. 3a). Examination of the colon at sacrifice, 45 days after the last DSS treatment, revealed a slightly (but non-significantly) reduced length in the 2% and 3% DSS groups (Additional Fig. 3b), and significant histological signs of persisting moderate inflammation of the distal colon for the same groups (Fig. 1d, Additional Fig. 3c).

The consequences for the intestinal microbiota were traced by 16S rRNA gene profiling over time (Fig. 1b). The first DSS pulse resulted in a transient reduction of microbiota alpha diversity in the 1% and 2% DSS groups (Additional Fig. 4). Intra-group variability temporarily increased in all groups, including the control group without DSS. Ten days after the last DSS pulse (T40), both intra-group variation and alpha diversity levels in individual rats had come back to pre-treatment levels. Together, the observations on host and microbiota constitute a first indication that DSS treatments lead to a perturbation of the ecosystem, after which the system appears to re-equilibrate.

**Fig. 2.**
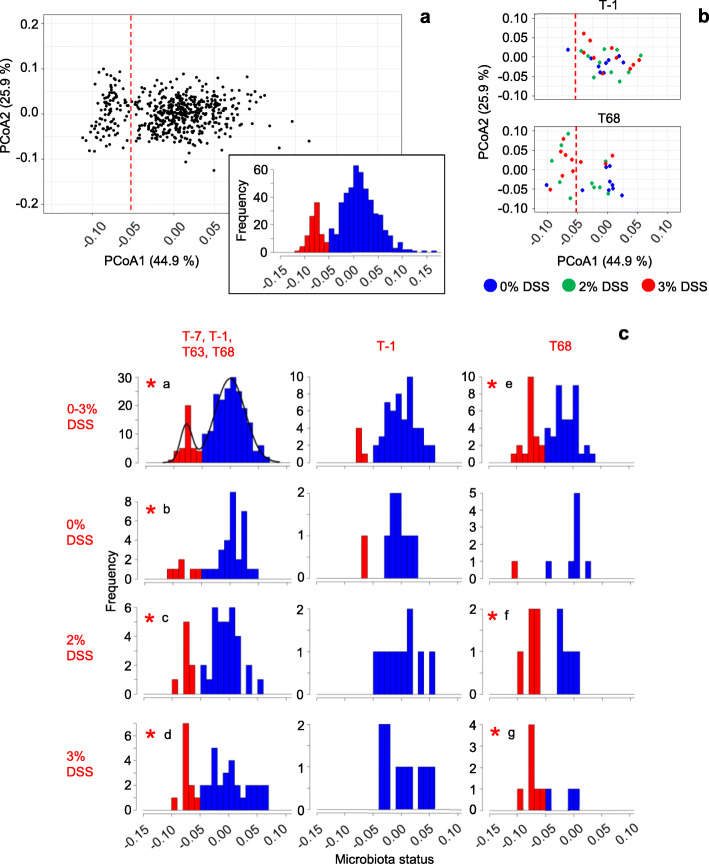
Two microbiota states. **a** Principal Coordinates analysis (PCoA) of ecological divergence between microbiota samples (Jensen-Shannon Divergence), based on OTU data aggregated at genus level. Each dot represents one intestinal microbiota sample. The analysis includes the data from all experimental groups, for T-7 up to T68 (*n* = 562 samples; cf Table 1). The dashed red line corresponds to the separation between the two normal distributions in the inset (frequency distribution of PCoA1 using the same samples) and in **c**. **b** Subsets of the data in **a**, for three experimental groups at T-1 and T68, respectively. Colors represent treatment groups as indicated. **c** Bimodal distribution of microbiota status. PCoA1 coordinates from the ordination plot in **a** as a measure of microbiota status are divided in categories with a range of 0.01, and the frequency of occurrence of each category is plotted. Top left: combined data from all groups (0% up to 3% DSS) for T-7, T-1, T63, and T68 (*n* = 232 samples). Bimodal graph overlay (density) and coloration according to the results of finite Gaussian mixture modeling using Mclust 5.4 [18]. Blue and red represent basal state and alternative state microbiota, respectively. Other plots: data for groups (% DSS) and time-points as indicated at the left and at the top of the figure, respectively. Coloration according to the bimodal distribution in the top left plot. Scale of the vertical axis is adjusted for each plot individually. *, bimodal (mixed normal) distributions according to Mclust 5.4 [18] when analyzing per-plot data (maximal value of the Bayesian Information Criterion (BIC), corroborated by bootstrap sequential Likelihood Ratio Testing (LRT): **a** *p* = 0.001 (cf Additional Table 3); **b** *p* = 0.004; **c** *p* = 0.032; **d** *p* = 0.027; **e** *p* = 0.01; **f** *p* = 0.04; **g** *p* = 0.03)

Principal coordinates analysis (PCoA) of the composition of microbiota samples illustrates the differentiating effects of successive DSS treatments on microbiota community structure beyond alpha diversity. In a first analysis of the combined data from all treatment groups for T-7 to T68, aggregated at the genus level, we find two microbiota types separated along the first PCoA axis (PCoA1; Fig. 2a, Additional Fig. 6). A closer examination of the data from the 0%, 2% and 3% DSS groups reveals that the microbiota from nearly all the rats in these groups belong to the same type at T-1 (i.e., before DSS treatment) (Fig. 2b). At T68 (38 days after the last DSS treatment), however, most of the microbiota in the 3% DSS group are of the alternative type, while nearly all microbiota from the 0% DSS control group are of the same type as at T-1. Microbiota from the 2% DSS group are equally distributed over the two types at T68.

These first observations are corroborated by a more detailed analysis where the PCoA1 coordinate of individual microbiota samples, which explains 44.9% of variation, is used as an indicator of microbiota status. Binning of microbiota samples with similar PCoA1 coordinates reveals a bimodal (mixed normal) frequency distribution of microbiota status when all data from time-points T-7, T-1, T63, and T68 are combined (Fig. 2c, top left), implying the existence of two distinct microbiota types. These microbiota types, or states, can be considered “alternative states”, as the external conditions (i.e., external to the rat) at these time-points are identical for all groups (no DSS present before T0 or after T31). A similar image is obtained when combining all data from T-7 up to T68, i.e., including samples obtained during the DSS treatments period (see inset in Fig. 2a; microbiota state attributions for individual rats at different time points are listed in Table 1). While the combined data (Fig. 2c, top left) provide the statistical power to ascertain bimodality of the distribution (Additional Table 3), the resulting framework is used to classify microbiota samples from subcategories (one treatment group, one time-point) through mapping on the full data distribution (Fig. 2c). Thus, most of the microbiota in the 3% DSS group appear to shift from one state (“basal state”, indicated in blue) at T-1 (i.e., before DSS treatment) to the alternative state (indicated in red) at T68 (Fig. 2c, bottom). In contrast, the microbiota in the 0% DSS control group essentially remain in the same basal state over time. In the 2% DSS group, half of the rats switched from one microbiota state to the other, resulting in a clear bimodal distribution at T68, within a group for which not only the external conditions at the time of analysis (T68) are identical, but also the history of external conditions. This observation further corroborates the conclusion of existence of alternative microbiota states.

**Table 1.** Microbiota-state time series

DSS	rat	T-7	T-1	T3	T13	T17	T27	T31	T40	T63	T68/75
1	48	1	1	1	1	1	1	nd	**2**	1	1A
0.25	26	1	1	1	1	1	**2**	**2**	**2**	1	1A
0	17	1	1	1	1	1	**2**	**2**	**2**	1	1A
0.25	21	1	1	1	1	1	1	1	**2**	1	1A
0	13	1	1	1	1	1	1	1	**2**	1	1A
0	19	1	1	1	1	1	nd	nd	1	1	1A
0.25	30	1	1	1	**2**	1	1	nd	1	1	1A
2	56	1	1	1	1	1	1	nd	1	1	1A
1	50	1	1	1	1	1	1	nd	1	1	1A
1	49	1	1	1	1	1	1	nd	1	1	1A
1	45	1	1	1	1	1	1	nd	1	1	1A
0.5	40	**2**	1	1	1	1	1	nd	1	1	1A
0.5	39	1	1	1	1	1	1	nd	1	1	1A
0	14	1	1	1	1	1	1	nd	1	1	1A
3	11	1	1	1	1	**2**	1	1	1	1	1A
0.25	28	1	**2**	**2**	1	1	1	1	1	1	1A
0	12	**2**	**2**	**2**	1	1	1	1	1	1	1A
0.5	34	1	**2**	1	1	1	1	1	1	1	1A
2	53	1	1	1	1	1	1	1	1	1	1A
1	46	1	1	1	1	1	1	1	1	1	1A
1	44	1	1	1	1	1	1	1	1	1	1A
0.5	38	1	1	1	1	1	1	1	1	1	1A
0.5	37	1	1	1	1	1	1	1	1	1	1A
0.5	36	1	1	1	1	1	1	1	1	1	1A
0.5	31	1	1	1	1	1	1	1	1	1	1A
0.25	29	1	1	1	1	1	1	1	1	1	1A
0.25	23	1	1	1	1	1	1	1	1	1	1A
0.25	22	1	1	1	1	1	1	1	1	1	1A
0	16	1	1	1	1	1	1	1	1	1	1A
0	15	1	1	1	1	1	1	1	1	1	1A
0	20	1	1	1	1	1	**2**	nd	**2**	**2**	1B
2	58	1	1	1	1	1	**2**	**2**	**2**	1	1B
2	60	1	1	1	1	1	1	nd	1	1	1B
2	54	1	1	1	1	1	**2**	**2**	1	1	1B
0.5	33	1	1	1	1	1	1	**2**	1	1	1B
3	65	1	1	1	1	1	1	1	1	1	1B
3	43	1	1	1	1	1	1	1	1	1	1B
1	42	1	1	1	1	1	1	1	1	1	1B
1	41	1	1	1	1	1	1	1	1	1	1B
3	70	1	1	1	1	1	**2**	nd	**2**	**2**	**2A**
0	18	**2**	1	1	1	**2**	**2**	**2**	**2**	**2**	**2A**
0.5	35	1	1	1	1	1	**2**	**2**	1	**2**	**2A**
2	57	1	1	1	1	1	**2**	**2**	**2**	1	**2A**
2	59	**2**	1	1	1	1	1	nd	1	1	**2A**
0.5	32	1	1	1	1	nd	**2**	**2**	1	1	**2A**
0.25	27	**2**	**2**	**2**	1	1	1	1	1	1	**2A**
0.25	25	1	**2**	1	1	1	1	**2**	1	1	**2A**
0.25	24	1	1	1	1	1	1	1	1	1	**2A**
3	64	1	1	1	1	1	nd	**2**	**2**	**2**	**2B**
3	68	1	1	1	1	1	**2**	**2**	**2**	**2**	**2B**
2	52	1	1	1	1	1	**2**	**2**	**2**	**2**	**2B**
2	51	1	1	1	1	1	1	**2**	**2**	**2**	**2B**
3	62	1	1	1	**2**	**2**	**2**	**2**	1	**2**	**2B**
3	69	1	1	1	1	1	1	nd	**2**	1	**2B**
3	67	1	1	1	1	1	**2**	**2**	**2**	1	**2B**
2	55	1	1	1	**2**	1	1	**2**	**2**	1	**2B**
1	47	**2**	1	1	1	1	**2**	**2**	1	1	**2B**
3	66	1	1	1	1	1	1	1	1	1	**2B**

Evolution of microbiota state with time is indicated for individual rats. *DSS* percentage of DSS used during treatment periods, *rat* rat number; T-7 to T63, time-points of fecal sampling; T68/75, T68 for fecal sampling and T75 for distal colon inflammatory status evaluation, respectively; *1* basal-state microbiota, *2* (bold script), alternative-state microbiota, *nd* not determined, *A* basal host inflammatory state, *B* alternative host inflammatory state (low-level inflammation). Rats (lines of the table) are grouped according to the host-ecosystem state at T68/75 (last column, cf Fig. 5), then by resemblance of microbiota-by-time profile from T27 onward

The same conclusion is reached after analysis of the data by an “enterotyping” approach [6], as an alternative to the PCoA approach described above. This approach resulted in the detection of three clusters (Additional Fig. 6a, b, d). Of these, cluster A roughly corresponds to the PCoA1-based alternative microbiota state 2, while clusters B and C together represent basal microbiota state 1 (Additional Fig. 6c). This observation is further illustrated by the juxtaposition of Table 1 (microbiota-state time series per rat) and the corresponding table showing cluster time series, where the alternative microbiota state 2 and cluster A show highly resembling distribution patterns (Additional Fig. 7).

**Fig. 3.**
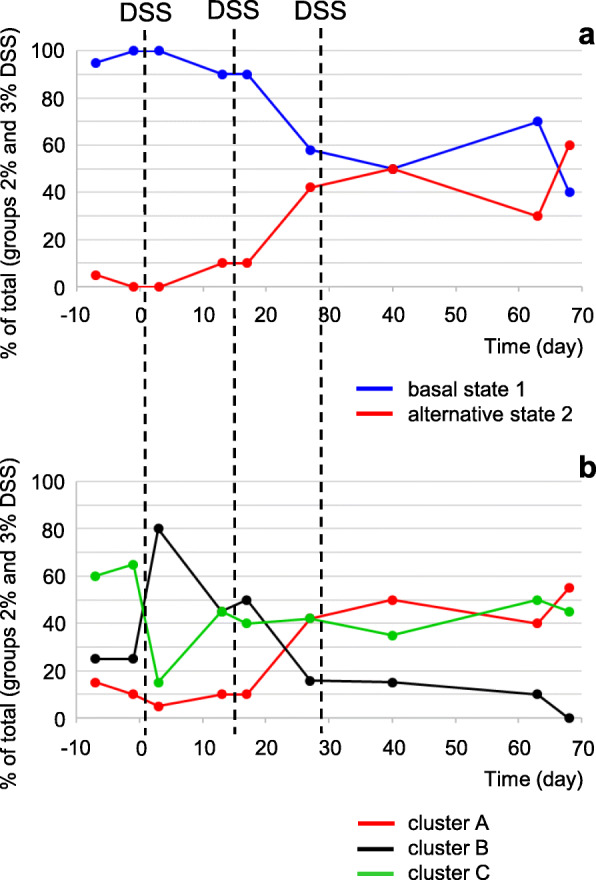
Evolution of microbiota states and enterotyping-based clusters with time. **a** Percentage of rats with PCoA1-based basal state or alternative state microbiota (cf Fig. 2). **b** Percentage of rats with enterotyping-based cluster A, B, or C microbiota. For both panels, percentage in groups treated with 2% or 3% DSS as indicated in Fig. 1. Sampling points as indicated in Fig. 1. Total number of rats sampled is 20 for each time point, except for T27 (19 rats). T31 was omitted because of missing data (only 15 rats sampled)

As a consequence, alternative microbiota state 2 and cluster A representation show a near identical development with time, as illustrated in Fig. 3 for the groups that had been treated with 2% or 3% DSS, where the highest numbers of stable state transitions are observed. Both microbiota state 2 and cluster A show a sharp increase between T17 and T27, i.e., in the period following the second DSS treatment. While the expansion of microbiota state 2 is directly mirrored by a contraction of microbiota state 1, the evolution of clusters B and C accompanying the increase of cluster A appears more complex. The apparent uneven equilibrium between the former two clusters at T0 is inversed by the first DSS treatment, and partially restored thereafter. After renewed perturbation by the second DSS treatment, cluster B declines and disappears by T68 while cluster C stabilizes at about two thirds of its initial level of representation. Reductions of both cluster B and cluster C representation hence mirror the raise of cluster A.

The results of the PCoA and enterotyping approaches thus lead to the same conclusion: DSS treatments induce a stable state transition, to PCoA1-based state 2 or the corresponding cluster A. Importantly, the results show that microbiota-state separations traverse treatment groups. Further analyses therefore focus on distinguishing microbiota states rather than treatment groups.

### Alternative microbiota-state occurrence is related to host inflammatory status

**Fig. 4.**
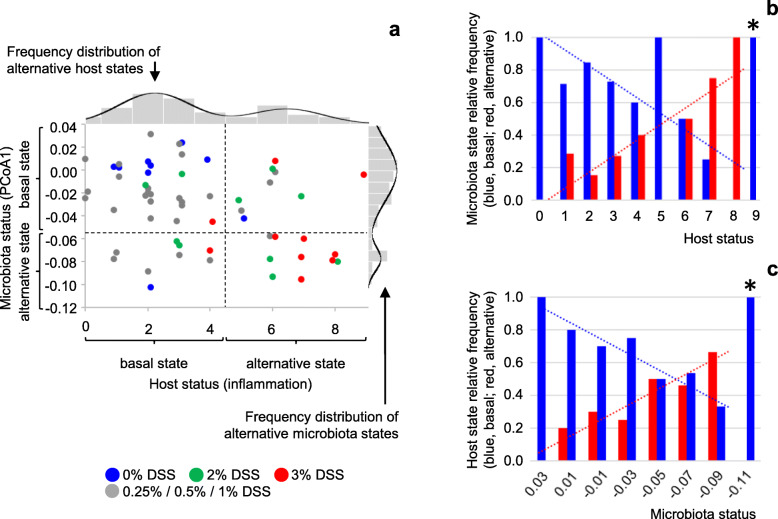
Microbiota status *versus* host inflammatory status. **a** Microbiota status at T68 (PCoA1 coordinate from Fig. 2a), plotted against host inflammatory status at T75 (histological score of the distal colon (Additional Tables 4 and 5); higher scores indicate higher levels of inflammation). Histological scores are integers; jitter has been added to improve visibility of individual data-points. Each dot represents one rat (*n* = 58). Colors represent treatment groups as indicated. The side-panel shows the frequency distribution and density curve of alternative microbiota states from Fig. 2c (top left: all groups). The top-panel shows the frequency distribution of alternative host states from Additional Fig. 8a, to which a density curve was added according to the results of finite Gaussian mixture modeling using Mclust 5.4 [18]. **b** Relative frequencies of alternative microbiota states as a function of host inflammatory status (distal colon histology score). Relative frequencies are calculated from the data in panel a. When separately analyzing the limited numbers of observations in subgroups (same histology score *H*), bimodal (mixed normal) distributions could be confirmed for subsets *H* = 2 (*p* =0.04) and *H* = 6 (*p* = 0.05) (finite Gaussian mixture modeling using Mclust 5.4 [18] with bootstrap sequential Likelihood Ratio Testing (LRT)). *, one observation only; each of the other categories represents from 3 to 13 observations. For host status 0 to 8, microbiota state distribution strongly correlates with host status (linear regression, *r*^2^ = 0.63, *p* = 0.01 (*F*-test)). **c** Relative frequencies of alternative host states as a function of microbiota status. Relative frequencies are calculated from the data in **a**. Microbiota status is expressed as PCoA1 coordinate (Fig. 2a) with binning in intervals of 0.02; the central value of each bin is indicated. *, one observation only; each of the other categories represents from 3 to 13 observations. For microbiota status categories − 0.09 to 0.03, host state distribution strongly correlates with microbiota status (linear regression, *r*^2^ = 0.91, *p* = 0.0009 (*F*-test))

**Fig. 5.**
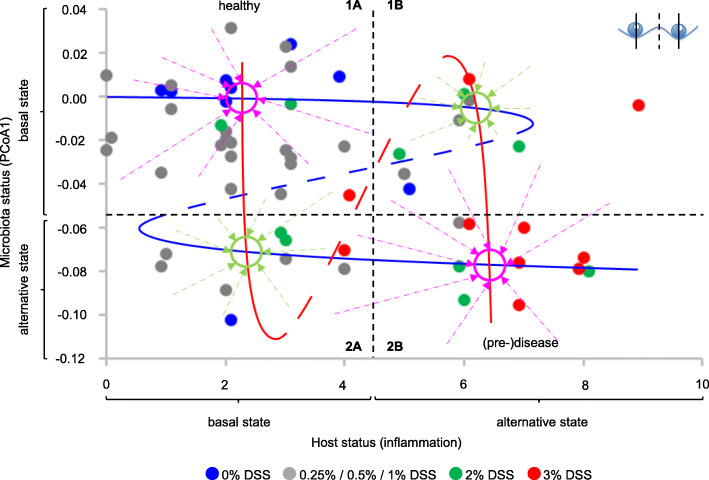
Model of the host-microbiota ecosystem. Microbiota status at T68 plotted against host inflammatory status at T75 (n = 58). The blue tentative Z-shape overlay characterizes alternative microbiota states (cf Fig. 1a). Continuous parts of the curve represent the cores of the alternative states, and are drawn in correspondence with the means of the frequency distributions in the side panel of Fig. 4a. The dashed diagonal represents the changing frontier between the two basins of attraction (a transition fold, see inset and Fig. 1a). A larger distance between a solid line and the dashed diagonal indicates a wider basin of attraction, resulting in a higher state stability as revealed by a higher relative frequency of observation (Fig. 4b). Approximate positions of the inflexion points (tipping points) are based on the frequency distributions of alternative microbiota states (Fig. 4b: change from two alternative states to one state). The red tentative S-shape overlay characterizes alternative host states. Continuous parts of the curve represent the cores of the alternative states, and are drawn in correspondence with the means of the frequency distributions in the top panel of Fig. 4a. Changing distances between solid lines and the dashed diagonal represent the observed changes in the relative frequencies of the two host states with microbiota status (Fig. 4c). Approximate positions of the inflexion points (tipping points) are based on the frequency distributions of alternative host states (Fig. 4c). Violet circles represent alternative stable states of the host-microbiota ecosystem (attraction points combining stable host and microbiota states); green circles represent fragile attraction points (less stable host and microbiota states, close to tipping points and frontiers between basins of attraction). Violet and green arrows illustrate predicted evolution of the ecosystem from different positions in the plot to the different attraction points (away from the dashed diagonals, towards the solid lines)

The results presented in Fig. 2c show that the alternative microbiota state (indicated in red) is more frequently observed after treatment with higher concentrations of DSS, which cause a phenotype of persistent moderate inflammation (Fig. 1d, Additional Fig. 3c). Hence, inflammatory status of the distal colon may be suspected as a driver of bimodality in microbiota composition. We therefore examined the relationship between distal colon inflammatory status (at T75) and microbiota state (at T68). The results of this analysis show that lower histology scores (less inflammation-related symptoms) are mostly associated with basal-state microbiota, but some are associated with alternative-state microbiota (Fig. 4a). The inverse is true for higher histology scores, which are predominantly associated with alternative-state microbiota while some are associated with basal-state microbiota. In fact, among animals with identical histology score, part present a basal state microbiota and part present an alternative state microbiota in nearly all cases, except at the extremes of the range of measured histology scores (Fig. 4b). This confirms that the two microbiota states can be considered alternative states (as opposed to condition-dependent states in a steep gradient (Fig. 1a)), represented by horizontal lines on the microbiota versus host status plot in Fig. 5 (horizontal lines of the tentative blue Z-shape overlay). The relative frequency of occurrence of each of the two alternative microbiota states is highly correlated with distal colon histology (Fig. 4b). This observation suggests that the frontier between the basins of attraction of the two states changes position as a function of host status, illustrated by the dashed diagonal part of the blue Z-shape overlay in Fig. 5 (cf Fig. 1a). The interrupted Z-shape is characteristic of a system with alternative stable states (Fig. 1a). State-transition can take place across the transition fold (the dashed diagonal), as a result of stochastic movements, or when the conditions on the horizontal axis change beyond the sharp bends in the curve (tipping points).

### Two alternative host states

Interestingly, distal colon histology score itself also shows a bimodal distribution, across all animals (Fig. 4a top panel, Additional Table 6) as well as within the 2% and 1% DSS treatment groups (Additional Fig. 8), implying two underlying host states. Six weeks after cessation of DSS treatments, microbiota composition can be suspected as a driver of sustained inflammation, and we therefore examined distal colon histology score as a function of microbiota status. Among animals with similar microbiota status, both host states can be observed in nearly all cases (Fig. 4c). The alternative host states are represented by vertical lines on the microbiota versus host status plot in Fig. 5 (vertical lines of the tentative red S-shape overlay). The relative frequencies of the two host states are highly correlated with microbiota status (Fig. 4c), suggesting a moving frontier between the basins of attraction of the two host states, illustrated by the dashed diagonal part of the red S-shape overlay. The interrupted S-shape, mirror image of the interrupted Z-shape described above regarding microbiota states, is in the same way characteristic of a system with alternative stable states.

### Alternative states of the host-microbiota ecosystem: a new model

Microbiota state thus appears to depend on host inflammatory status, and host state on microbiota status, consistent with the growing body of evidence for reciprocal impact and the increasing mechanistic understanding of the processes involved. Both microbiota status and host status show distributions coherent with the existence of alternative stable states. The combination of these distributions of host and microbiota alternative stable states yields new insights in the alternative states of the host-microbiota ecosystem as a whole and provides a theoretical framework to predict its behavior, as illustrated in the conceptual model presented in Fig. 5.

This model predicts four attraction points for the host-microbiota ecosystem, at the intersections of stable microbiota states and stable host states. Two of these (indicated in violet in Fig. 5) can be described as stable “healthy” (top left) and “(pre-)disease” (bottom right) states, combining basal microbiota and host states or alternative microbiota and host states, respectively. The two others (indicated in green in Fig. 5) are expected to show less resilience as they are situated closer to tipping points and transition folds, both with respect to microbiota state and with respect to host state, and therefore be less stable. These attraction points can be regarded as risk situations because small (stochastic or environment-driven) changes in host or microbiota status can provoke a switch to the more stable “(pre-)disease” state. At the same time, these attraction points can also be regarded as therapeutic opportunities because small adjustments in host or microbiota status could also trigger a return to the more stable “healthy” state.

**Fig 6.**
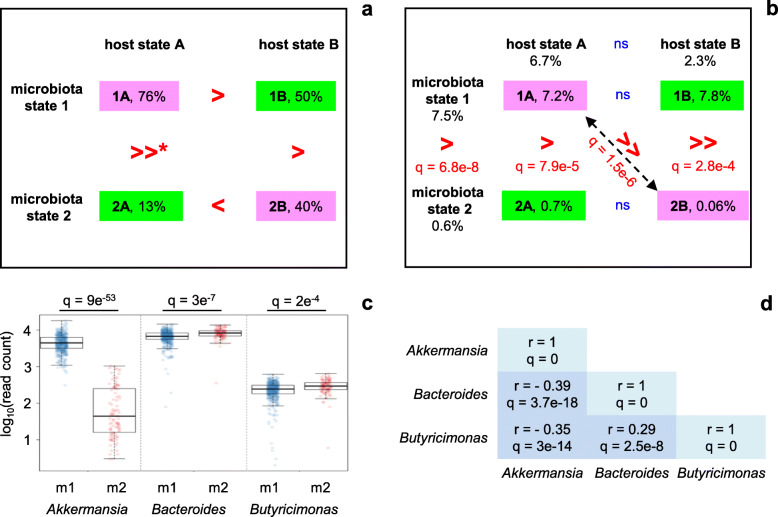
Characterization of attraction points in the host-microbiota ecosystem model. **a** Stability of attraction points. For each of the quadrants 1A, 1B, 2A, and 2B from Fig. 5 the percentage of animals presenting a stable microbiota is indicated. Stable microbiota is defined as showing the same state (either basal state 1 or alternative state 2) at four consecutive sampling time-points (T31, T40, T63, T68; i.e., from just after the last DSS treatment onward). >, >>, and < signs are read from left to right and from top to bottom. *p = 0.003 (Fisher exact test). **b** Relative abundance of *Akkermansia* in different microbiota, host, and ecosystem states. Abundance is expressed as percentage of total number of sequence reads; median values for the rats in each state at T68 are represented. Statistically significant differences are indicated in red. Differences between ecosystem states (1A, 1B, 2A, 2B) were analyzed using a Kruskal-Wallis test with post hoc Dunn’s test and Holm correction for multiple comparisons; differences between microbiota states (1, 2) or host states (A, B) were analyzed using a Wilcoxon test with FDR adjustment. **c** *Akkermansia*, *Bacteroides* and *Butyricimonas* distributions in microbiota states 1 (m1) and 2 (m2). Combined data from T-7 to T68 (*n* = 463 samples for microbiota state 1, *n* = 99 samples for microbiota state 2 (cf Table 1)); each dot represents one intestinal microbiota sample. Abundance is expressed as number of sequence reads on a total of 38,000. Only genera for which the median abundances in the two microbiota states differ at least 1.2-fold with *q* < 0.05 (Wilcoxon test with FDR adjustment) are presented. **d** Spearman correlation (*r*) between *Akkermansia*, *Bacteroides*, and *Butyricimonas* abundances. *q* values, after Holm correction for multiple comparisons

Regarding microbiota state, the prediction of differential stability of the four attraction points appears to be supported by time course data from individual rats, although many more animals would be needed to conclude. The data presented in Table 1 show that the microbiota of rats that end up in the upper left part of Fig. 5 at T68 (quadrant 1A; basal microbiota state 1, basal host state A) are much more frequently found in this same microbiota state 1 at four consecutive sampling time-points (T31, T40, T63, T68; i.e., from just after the last DSS treatment onward until the end of the experiment) than the microbiota of rats in the lower left part of the figure (quadrant 2A; alternative microbiota state 2, basal host state A) are found in microbiota state 2 (their state at T68) (76% vs 13%, respectively (Fig. 6a)). This significant difference (Fisher exact test, *p* =0.003) is in agreement with the prediction that host-microbiota state 1A would be more resistant to microbiota state changes than state 2A. Two other tendencies support the model without reaching statistical significance. The microbiota from rats in quadrant 1A appear to be more stable than the microbiota from rats in quadrant 1B (basal microbiota state 1, alternative host state B) (76% of rats showing the same microbiota state at T31, T40, T63 and T68 vs 50%, respectively), and the microbiota from rats in quadrant 2B (alternative microbiota state 2, alternative host state B) appear to be more stable than the microbiota from rats in quadrant 2A (40% vs 13%, respectively). Only the comparison between quadrants 1B and 2B (50% vs 40% stability, respectively) does not support the model, although in view of the small number of observations this smallest difference among all comparisons made does not contradict the model either.

The data in Table 1 also confirm that bimodal microbiota distributions at T68 are not due to the prior existence of two static subpopulations of rats with different microbiota compositions. Longitudinal analysis of the microbiota component shows that the microbiota of a given rat can sometimes switch from one state to another and back again, including before DSS treatment or in the control group without DSS treatment, without questioning the long-term stability of the alternative states. Hence, DSS treatment does not create a new microbiota state, but rather affects the transition between, or the stability of, existing microbiota configurations.

### Characterization of alternative ecosystem states

A comparison of relative bacterial genus abundances between the two microbiota states 1 and 2 reveals that the alternative state (state 2) is characterized by a strongly reduced abundance of *Akkermansia* (Fig. 6b, c) and slightly increased abundances of *Bacteroides* and *Butyricimonas* (Fig. 6c). Variations in the relative abundance of the latter two genera are anti-correlated with variations in *Akkermansia* abundance (Fig. 6d). As expected, a strongly reduced abundance of *Akkermansia* also constitutes the distinguishing feature separating enterotyping-based cluster A (which corresponds to PCoA-based microbiota state 2) from the other clusters (Additional Fig. 6d). An even stronger difference in relative abundance of *Akkermansia* between ecosystem states 1A (basal microbiota and host states) and 2B (alternative microbiota and host states, with moderate inflammation) (Fig. 6b) is consistent with earlier reports of an inverse association of *Akkermansia muciniphila* with (low-grade) inflammation in humans [19].

The alternative, (pre-)disease, host state (state B (Fig. 5)) is characterized by moderate levels of (in order of contribution to the histology scores) mononuclear cell infiltration, epithelial atrophy, edema and ulceration (Additional Table 5).

## Discussion

Alternative stable states have been described for various ecosystems, ranging from seemingly very simple model systems [20] to very complex systems responding to global climate change [21]. Depending on the level of complexity and manageability of the system, analytical approaches have involved the experimental manipulation of key parameters (“external conditions”) or are, inevitably when working at the scale of climate change, oceans or rain forests, purely descriptive in nature. The latter approach depends on the study of many parallel ecosystems and involves the justification of the choice of a variable from the available metadata as the condition parameter, the establishment of a correlation between this parameter and system state, and the demonstration of multi-modal distributions of system state at various values of this parameter [21, 22].

The present study borrows from both approaches, with two levels of analysis. At the first level, the ecosystem under study is the rat intestinal ecosystem, in fact 58 parallel ecosystems (58 rats), with its two components, microbiota and host. We control the external conditions and apply a transient alteration of one external parameter, the amount of DSS in the drinking water, to different levels in different experimental groups. Thirty-eight or 45 days later, when the external conditions are identical for all ecosystems, distributions of both microbiota and host inflammatory status are bimodal, implying two underlying states for each component, and potentially four for the host-microbiota ecosystem as a whole. Inflammation, here induced by DSS treatment, appears to either stabilize an alternative microbiota state resulting from spontaneous state-transition, to facilitate additional transitions by moving the system to a fragile state more prone to spontaneous transition, or a combination of the two (Additional Fig.9). Low-level inflammation (alternative host state) and alternative microbiota state remain stable up to at least 6 weeks after cessation of DSS treatments (i.e., up to the end of our experiment).

Our observations converge to the conclusion that alternative stable states exist in the rat intestinal ecosystem. They complement and extend the only earlier study that investigated the existence of alternative stable states in the (human) intestinal microbiota. Compared with this study [23], the use of a rat model allowed us to induce a state transition and control external conditions, an essential prerequisite to distinguish alternative ecosystem states from different states that can be attributed to different conditions in our first level of analysis (Fig. 1a). In addition, our study directly associates microbiota state and host inflammatory status, thus extending the description of alternative stable states to include the microbiota as well as the host component of the ecosystem [15]. Our results show the existence of host-microbiota ecosystem states rather than independent and dissociated microbiota and host states. The interplay between microbiota and host could account for the maintenance of stable equilibria (alternative states), as well as for a rapid switch to an alternative state (as observed in Fig. 3), a “catastrophic transition” [14], upon perturbation beyond a tipping point, a key feature of critical transition. This rapid transition could be brought about by the triggering of a vicious circle of mutually reinforcing events, where microbiota alteration promotes inflammation and in return, inflammation promotes sustained alteration of the microbiota (Additional Fig. 1) [15]. Future developments may allow to document early warning signals of transitions to come [24].

In order to fully appreciate the significance of the existence of alternative stable states in the intestinal ecosystem, we decided to take our analyses one step further. Therefore, at a second level of analysis, we take an inside view in the host-microbiota ecosystem and study each component, microbiota and host, separately, assuming that the state of each component depends on the status of the other component, which in this case can be regarded as an external condition. The assumption is a knowledge-based assumption, warranted by the overwhelming evidence of reciprocal impact of the intestinal microbiota and the host reported in the literature. This interdependency is clearly corroborated by our results: we identify two alternative stable microbiota states, and the frequency of occurrence of each is associated with the inflammatory status of the host. We also identify two alternative stable host states, and the frequency of occurrence of each is associated with the status of the microbiota.

**Fig. 7:**
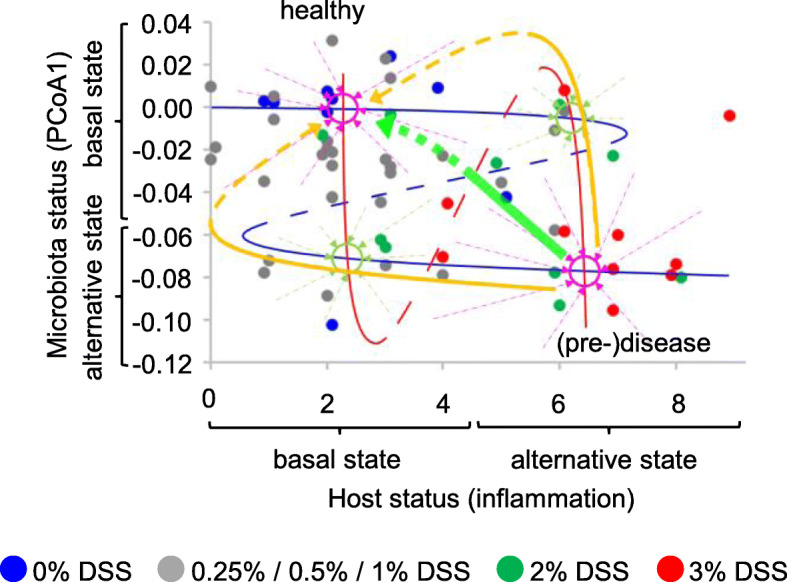
(Pre-)disease remediation strategies. Host-microbiota ecosystem model from Fig. 5. Yellow and green arrows show predicted requirements (solid lines) and outcomes (dashed lines) for disease remediation strategies based on host inflammatory status management (yellow line, bottom left), microbiota management (yellow line, top right), or combined host and microbiota management (green line). See main text for details

Consequently, the host-microbiota ecosystem state can be described as a function of two bimodal parameters, microbiota state and host state, providing new insights in its stability, be it in a healthy, a pre-disease, or a disease state, and a new theoretical framework to predict its behavior and guide therapeutic or prevention strategies. The model presented in Fig. 5 is to our knowledge unique in its kind and could, if applicable to humans, explain why chronic inflammatory diseases (or pre-disease states) are extremely difficult if not impossible to cure using conventional treatments that only target inflammation symptoms. For example, to return from the “(pre-)disease” state to the “healthy” state using anti-inflammatory drugs, one would have to reduce the host inflammatory tone to close to zero in order to bypass the tipping point of the microbiota state curve (Fig. 7, bottom left, yellow line). This objective may be difficult to reach because of the attraction point (indicated in green) encountered on the way. Moreover, if the system is pulled back to this attraction point, it has a high risk of falling back to the (pre-)disease state, as described in the results section. Clinically speaking it is also delicate to totally abrogate immune responsiveness, as it would make the ecosystem sensitive to infections. If one would act solely on the microbiota, using bacterial complements, one would have to push the system all the way up to the top of the figure in order to bypass the tipping point of the host state curve (Fig. 7, upper right, yellow line), at the risk of being caught by the green attraction point present on this path and ultimately falling back to (pre-)disease. In fact, these predicted outcomes very much resemble clinical experience in the treatment of inflammatory bowel diseases (IBD) using anti-inflammatory treatments or probiotics, or even surgical removal of intestinal segments, where success rates are low, chances of relapse are high, and inter-individual variability is important.

In contrast, the model predicts that restoration of health would be much easier accomplished through a combination of anti-inflammatory treatments and microbiota management (Fig. 7, green arrow). If the system can be pushed beyond the crossing of the transition folds of host states and microbiota states (the red and blue dashed lines, respectively), it is predicted to evolve to the healthy state without further action needed. Simultaneous action on host and microbiota would thus require less effort on each of these parameters to initiate a return to a healthy state, and this may make the difference between possible and not possible, curable and not curable.

While we expect this principle to hold in different situations, the shape of the model presented in Fig. 5 likely depends on factors like the richness and composition of the microbiota and/or external conditions such as diet and other life-style related parameters. In order to situate an individual in the model, and predict the risk of transition to a less favorable state or develop a personalized strategy of prevention or cure, one would thus need the right “reference map”, based on observations made in individuals with similar metadata. In the present study, a switch to a fiber-free diet was fully integrated in the design of the model, aiming to induce a low richness, less robust microbiota [25]. The resulting 25% reduction in the number of OTUs is comparable to that observed, for example, in healthy inhabitants of US metropolitan areas compared to healthy Amerindians from a rural area in Venezuela [26], which has tentatively been attributed to Western lifestyle, and notably diet. The diet switch was also expected, and indeed observed, to induce a loss of *Prevotella* [27], and a rise in mucolytic *Bacteroides* and sulfate reducing *Desulfovibrio*, both favoring a more pro-inflammatory context (Additional Fig. 2).

Taken together, our results confirm the hypothesis of existence of alternative stable states in the rat intestinal ecosystem. It is tempting to believe that our model applies to the human intestinal ecosystem and microbiota enterotypes, which have been linked to differing (low-grade) inflammation levels in both mice [28] and humans. In a context where microbiota composition, and more generally intestinal ecosystem state, are increasingly linked to health or disease, the notion of alternative stable states may be very important as it could condition the design of innovative strategies to be used to maintain or restore symbiosis in order to prevent or cure disease. Models like the one presented in this study could, if validated in humans, be used to elaborate adequate preventive or therapeutic strategies.

## Conclusions

In this study, we provide the first formal experimental proof for the existence of alternative stable states in the rat intestinal ecosystem, taking both the microbiota and the host inflammatory status into consideration. Our results show the existence of host-microbiota ecosystem states rather than independent and dissociated microbiota and host states. The results lead us to propose a conceptual model providing new insights in the interplay between host inflammatory status and microbiota status. These new insights call for innovative therapeutic strategies to cure (pre-)disease. If validated in humans, our model will support diagnosis, choice of therapeutic strategy, and monitoring of progress during therapy.

The results presented in this study provide a strong rationale for the application of combinatorial preventive and therapeutic strategies, targeting host and microbiota, in chronic immune-mediated diseases.

## Methods

### Study design

The aim of this study was to examine the existence of alternative stable states in the rat intestinal ecosystem. For this purpose, sixty conventional SPF Fischer 344 male rats, 5 weeks old, were obtained from Charles River Laboratories, Italy, and housed in individual cages in a conventional facility at INRAE (*Unité Expérimentale d’Infectiologie des Rongeurs et Poissons*) where they received a standard chow diet (Additional Table 1, diet 1). (In the end, data from 58 rats were analyzed; see bioinformatics paragraph below.) After 10 days (T-49), feces were harvested, total DNA extracted, and microbiota composition evaluated by qPCR targeting the *Bacteroides*/*Prevotella* group or “all bacteria” as described in reference [29]. At T-31, animals were assigned to 6 experimental groups (10 rats per group), randomizing microbiota composition (% *Bacteroides/Prevotella* group at T-49), cage occupancy at the provider, affiliation (litter), and body weight at T-31 (Additional Fig. 10). From T-31 onward the chow diet was replaced by a diet without crude fiber, with a slightly changed macronutrient composition (Additional Table 1, diet 2). Groups received different doses (0%, 0.25%, 0.5%, 1%, 2%, or 3% w/v) of Dextran Sodium Sulfate (DSS; MP Biomedicals, MW 36,000–50,000) in autoclaved drinking water over three periods of three days between T0 and T30 (Fig. 1b). At the end of the experiment, rats were euthanized by cervical dislocation.

### Fecal microbiota 16S rRNA gene profiling

Fresh fecal samples were collected at regular intervals. Total DNA was extracted according to Costea et al. [30] (protocol #1). DNA integrity was assessed using a Fragment Analyzer (Agilent Technologies); DNA concentration was determined by Qubit (Invitrogen) and Nanodrop (Thermo Scientific). For each sample, microbiota composition was assessed by Miseq sequencing targeting the V3–V4 region of the bacterial 16S rRNA gene. Samples were prepared according to the Illumina protocol, using forward primer TCGTCGGCAGCGTCAGATGTGTATAAGAGACAGCCTACGGGNGGCWGCAG and reverse primer GTCTCGTGGGCTCGGAGATGTGTATAAGAGACAGGACTACHVGGGTATCTAATCC [31] for amplicon PCR (25 cycles).

### Host inflammatory status

At sacrifice, Swiss rolls were prepared from the distal part of the colon for histological evaluation of host inflammatory status. Hematoxylin eosin saffron (HES) and Periodic acid Schiff (PAS) stained slides were prepared at the histology facility @BRIDGe (GABI, INRAE, AgroParisTech, Paris-Saclay University). Inflammatory status was evaluated by a veterinary histopathologist in a blinded procedure using the criteria provided in Additional Table 4. Scores for individual criteria (Additional Table 5) were totaled to provide one final score per rat.

### Bioinformatics and statistical analysis

16S rRNA gene sequence analyses were performed using QIIME [32] (v. 19) and PhyloSeq [33]. After quality filtering, sequences of all samples were randomly sampled to keep 38,000 sequences per sample. Sequences were clustered into operational taxonomic units (OTUs; 97% identity threshold) using VSEARCH [34], and representative sequences for each OTU were taxonomically assigned using the SILVA database [35] (v. 119). Two rats were removed from the analysis, as they showed a significantly lower weight from the beginning and did not catch up with other rats before DSS treatments. Bimodality of microbiota and host status distributions was evaluated using Mclust 5.4 [18]. Non-parametric two-sided tests were used for taxonomic comparisons between microbiota states as specified in main text and figure legends. Enterotyping [6] was performed as described in https://enterotype.embl.de/enterotypes.html.

## Supplementary information

**Additional file 1 : Fig. 1**. Alternative stable states and critical transition in the gut microbiota - host symbiosis. Alternative stable states representing health (symbiosis, left) or (pre-) disease (altered symbiosis, right). The circle in the middle represents a vicious circle of self-enhancing deterioration of symbiosis, leading to critical transition to an alternative stable state of altered symbiosis. Adapted from reference [15].

**Additional file 2 : Fig. 2**. Effect of diet shift on microbiota composition. Panel a, Time course of relative abundance (number of sequence reads on a total of 38,000) of selected bacterial genera, before and after diet shift at T-31 (cf Additional Table 2). Colored lines each represent one rat. Dashed lines connect median values. Panel b, Abundance distributions of selected *Lactobacillus* species before (T-34) and after (T-20) diet shift (n=10). Abundance is expressed as number of sequence reads on a total of 38,000. Each dot represents one rat. P-values, Wilcoxon test.

**Additional file 3 : Fig. 3**. Effect of DSS treatments on the host. Panel a, Development of rat bodyweight from the start of the first DSS treatment (T0) onward, relative to weight at T0. Curves represent means of treatment groups. Colors represent treatment groups as indicated. Red horizontal lines at the bottom of the figure indicate DSS treatment periods. Statistically significant differences are observed between 0% and 3% DSS groups at T2, T3, T16, T17, T18 and T19 (p < 0.05; Kuskal-Wallis test with Dunn’s post test, adjusted for multiple testing). Differences at other time-points or between other treatment groups and the 0% DSS group were not significant. Panel b, Colon length at T75 (45 days after last DSS treatment), in different treatment groups. Each mark represents one rat. Horizontal lines indicate median values. Differences between treatment groups are not statistically significant (Mann Whitney test). Panel c, Distal colon histology scores (total inflammation scores from Additional Table 5) at T75 in different treatment groups. Each mark represents one rat. Horizontal lines indicate median values. Statistically significant differences are observed between 0% and 2% DSS groups (p=0.0068) and between 0% and 3% DSS groups (p=0.0001) (Mann Whitney test).

**Additional file 4 : Fig. 4**. Effect of DSS treatments on microbiota diversity. Microbiota alpha diversity (Shannon index) at time-points indicated at the bottom of the figure. Each dot represents one rat. Colors represent treatment groups as indicated. Red arrows indicate DSS treatments (T0 to T2, T14 to T16, and T28 to T30, respectively). At T3, alpha diversity was significantly lower in the 0.5%, 1% and 2% DSS groups than in the 0% DSS control group (p < 0.01; Kuskal-Wallis test with Dunn’s post test, adjusted for multiple testing). No differences between treatment groups were observed at T-1, nor at T68. In the 1% and 2% DSS groups, alpha diversity was lower at T3 than at T-1, while the opposite was true in the 0% DSS control group (p < 0.05; Wilcoxon test). None of the treatment groups showed significant differences in alpha diversity between T-1 and T68 (Wilcoxon test).

**Additional file 5 : Fig.5**. Principal coordinates analysis. 3rd and 4th axes of the principal coordinates analysis of microbiota data shown in Fig. 2a.

**Additional file 6 : Fig. 6**. Enterotyping. Microbiota data from T-7 to T68 were aggregated at genus-level, filtered for “unknown” and “uncultured” attributions, and analyzed using the clustering approach described in [6] (“enterotyping”). Panel a, clustering score (Calinski-Harabasz index [36]) as a function of the number of clusters shows a maximum at 3 clusters. Panel b, clustering with 3 clusters. Panel c, correspondence between clusters in panel b and PCoA1-based microbiota states from Fig. 2 (1, basal state; 2, alternative state). Numbers indicate number of samples (Table 1) in each category. Microbiota state 2 roughly corresponds to cluster A. Panel d, Akkermansia, *Phascolarctobacterium* and *Bacteroides* distributions in clusters A, B and C. Combined data from T-7 to T68; each dot represents one intestinal microbiota sample. Abundance is expressed as number of sequence reads on a total of 38,000. Only genera for which the median abundances in the two microbiota states differ at least 1.2-fold with q < 0.05 (Wilcoxon test with FDR adjustment) are presented. The table presents q-values for pairwise comparisons of relative abundances between clusters, for each of the three species (Kruskal-Wallis test with posthoc Dunn’s test and Holm correction for multiple comparisons).

**Additional file 7 : Fig. 7**. Correspondence between enterotyping-based clusters and PCoA1-based microbiota states. Juxtaposition of Table 1 (from main text, left) showing PCoA1-based microbiota states as a function of time for each rat, and the corresponding table showing enterotyping-based clusters from Additional Fig. 6b (right). Alternative microbiota state 2 and cluster A are highlighted. Rats (lines of the table) are grouped according to the host-ecosystem state at T68/75 (last column of Table 1, cf Fig. 5), then by resemblance of microbiota-by-time profile from T27 onward (cf Table 1).

**Additional file 8 : Fig. 8**. Two host states. Panel a, Host inflammatory status (distal colon histology score) distribution over all experimental groups (0% up to 3% DSS). Panel b, Distal colon histology score distributions for groups treated with 1, 2, or 3% DSS, and untreated control group. Colors represent treatment groups as indicated. Bimodal distributions are observed when data from all groups are combined (panel a, Additional Table 6), and within the 2% and 1% DSS groups (panel b).

**Additional file 9 : Fig. 9**. State transitions in the host-microbiota ecosystem. Host-microbiota ecosystem model from Fig. 5. Possible trajectories of the host-microbiota ecosystem from a “healthy” state to a “(pre-) disease” state, comprising a microbiota state-transition and a host inflammatory state-transition, are indicated by the three filled arrows. These transitions may be sequential (upper and lower arrows) or simultaneous (diagonal arrow).

**Additional file 10 : Fig. 10**. Constitution of experimental groups. Panel a, Composition of experimental groups regarding microbiota composition at T-49 (*Bacteroides*+*Prevotella* group as percentage of total bacteria, determined by qPCR), cage occupancy at the provider, and affiliation (litter). % DSS indicates experimental groups (DSS treatments between T0 and T30). Panel b, Correlation between *Bacteroides*+*Prevotella* content determined by qPCR and determined by MiSeq on the same DNA samples (Spearman's rank correlation: Rho = 0.77, p < 0.05). Panel c, Composition of experimental groups regarding body weight at T-31. No significant differences were observed between groups (ANOVA, p = 0.7).

**Additional file 11 : Table 1**. Diet composition. Description of diets used.

**Additional file 12 : Table 2**. Effect of diet shift on microbiota composition. Differential median relative abundances before (T-34) and after (T-20) diet shift for selected bacterial genera.

**Additional file 13 : Table 3**. Bimodal distribution of microbiota status. Statistical support for bimodal distribution of microbiota status (implying two microbiota states).

**Additional file 14 : Table 4**. Distal colon histology scoring criteria. Scoring criteria for evaluation of inflammatory status.

**Additional file 15 : Table 5**. Distal colon histology scores.

**Additional file 16 : Table 6**. Bimodal distribution of host inflammatory status. Statistical support for bimodal distribution of host inflammatory status (implying two host states).

## Data Availability

16S rRNA gene sequence data are available on NCBI under BioProject PRJNA549797. All other data are included in this article and its supplementary information files.
