# Supplementary figures and images for: Alternative stable states in the intestinal ecosystem: proof of concept in a rat model and a perspective of therapeutic implications
Source: Microbiome. 2020 Nov 6;8:153. doi: 10.1186/s40168-020-00933-7 (PMC7646066; doi:10.1186/s40168-020-00933-7)

## Slide 1
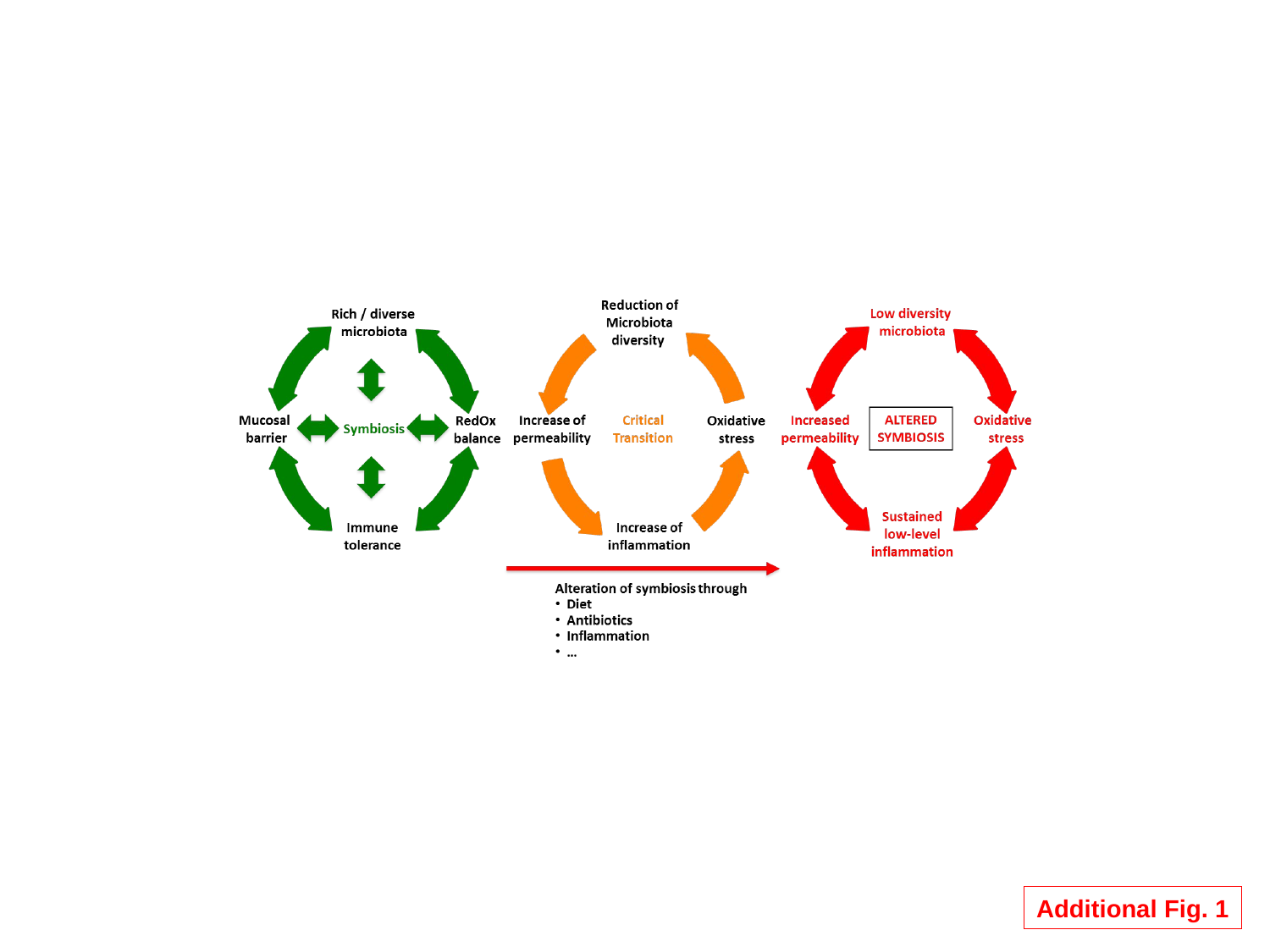

Additional Fig. 1

Supplement: Supplementary file 2 — Additional file 1 : Fig. 1. Alternative stable states and critical transition in the gut microbiota - host symbiosis. Alternative stable states representing health (symbiosis, left) or (pre-) disease (altered symbiosis, right). The circle in the middle represents a vicious circle of self-enhancing deterioration of symbiosis, leading to critical transition to an alternative stable state of altered symbiosis. Adapted from reference [15]. [file 40168_2020_933_MOESM1_ESM.pptx]

## Slide 1
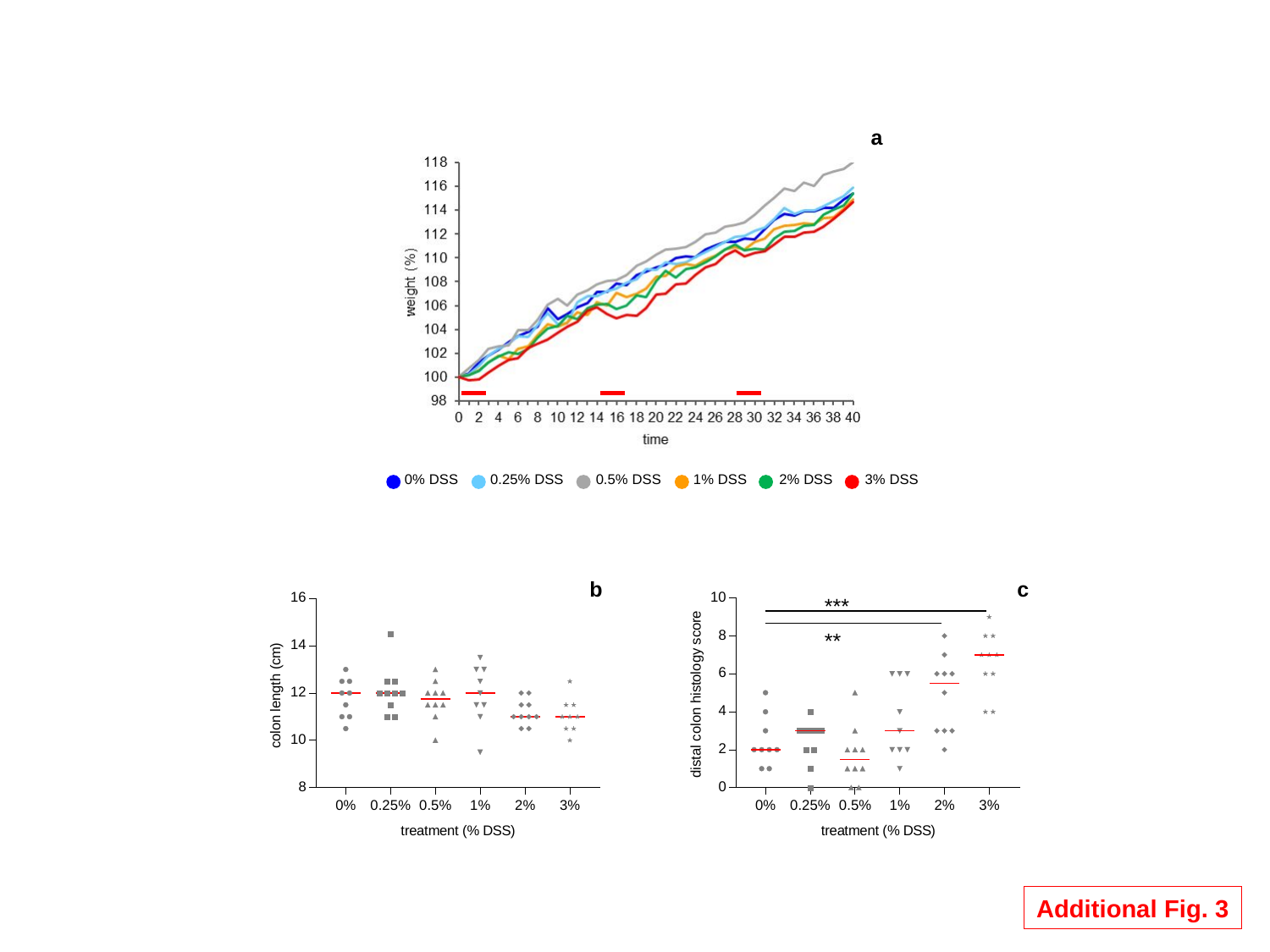

a
0% DSS 0.25% DSS 0.5% DSS 1% DSS 2% DSS 3% DSS
b
c
***
**
Additional Fig. 3

Supplement: Supplementary file 4 — Additional file 3 : Fig. 3. Effect of DSS treatments on the host. Panel a, Development of rat bodyweight from the start of the first DSS treatment (T0) onward, relative to weight at T0. Curves represent means of treatment groups. Colors represent treatment groups as indicated. Red horizontal lines at the bottom of the figure indicate DSS treatment periods. Statistically significant differences are observed between 0% and 3% DSS groups at T2, T3, T16, T17, T18 and T19 (p < 0.05; Kuskal-Wallis test with Dunn’s post test, adjusted for multiple testing). Differences at other time-points or between other treatment groups and the 0% DSS group were not significant. Panel b, Colon length at T75 (45 days after last DSS treatment), in different treatment groups. Each mark represents one rat. Horizontal lines indicate median values. Differences between treatment groups are not statistically significant (Mann Whitney test). Panel c, Distal colon histology scores (total inflammation scores from Additional Table 5) at T75 in different treatment groups. Each mark represents one rat. Horizontal lines indicate median values. Statistically significant differences are observed between 0% and 2% DSS groups (p=0.0068) and between 0% and 3% DSS groups (p=0.0001) (Mann Whitney test). [file 40168_2020_933_MOESM3_ESM.pptx]

## Slide 1
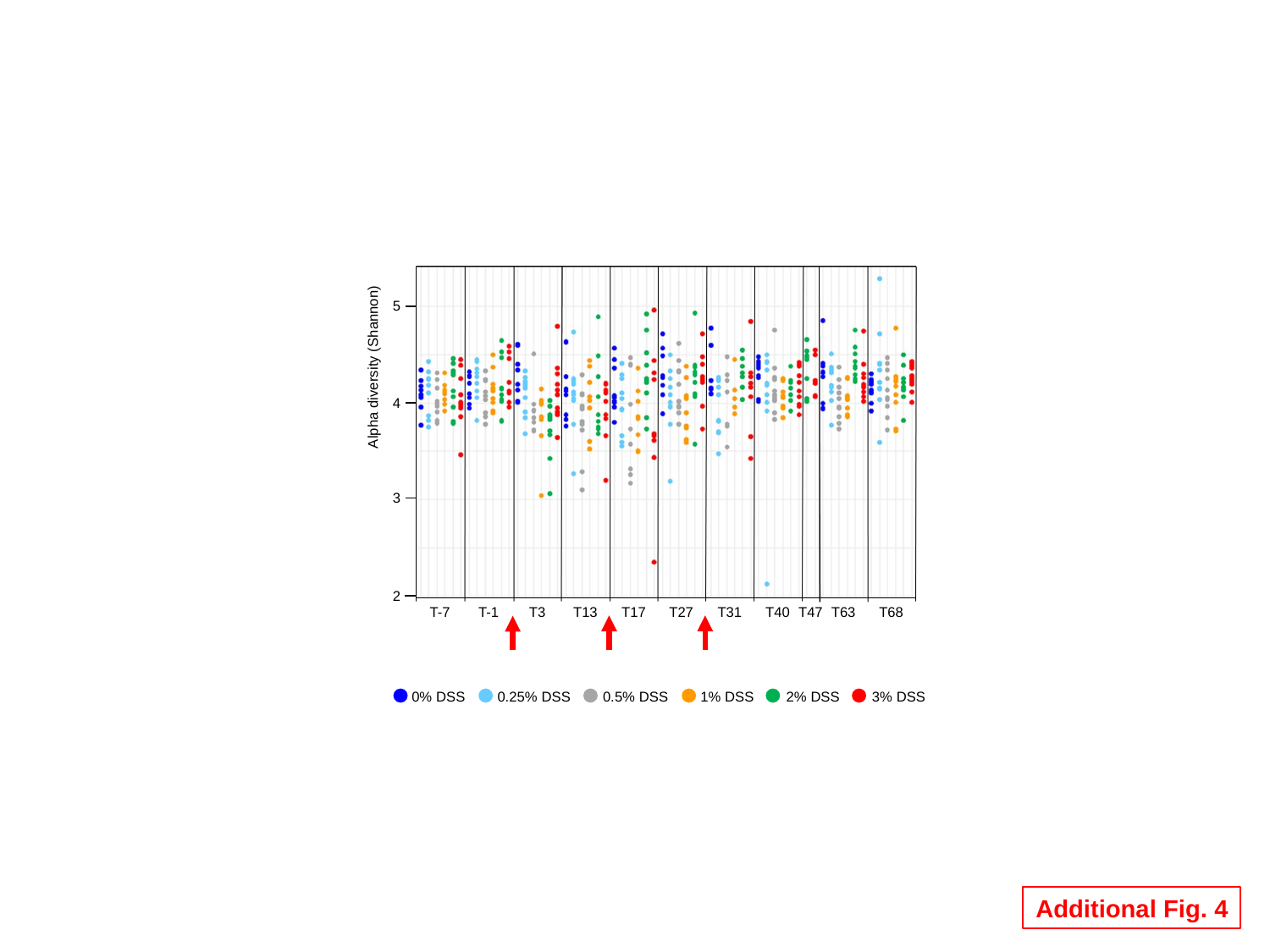

5
Alpha diversity (Shannon)
4
3
2
T-7
T-1
T3
T13
T17
T27
T31
T40
T47
T63
T68
0% DSS 0.25% DSS 0.5% DSS 1% DSS 2% DSS 3% DSS
Additional Fig. 4

Supplement: Supplementary file 5 — Additional file 4 : Fig. 4. Effect of DSS treatments on microbiota diversity. Microbiota alpha diversity (Shannon index) at time-points indicated at the bottom of the figure. Each dot represents one rat. Colors represent treatment groups as indicated. Red arrows indicate DSS treatments (T0 to T2, T14 to T16, and T28 to T30, respectively). At T3, alpha diversity was significantly lower in the 0.5%, 1% and 2% DSS groups than in the 0% DSS control group (p < 0.01; Kuskal-Wallis test with Dunn’s post test, adjusted for multiple testing). No differences between treatment groups were observed at T-1, nor at T68. In the 1% and 2% DSS groups, alpha diversity was lower at T3 than at T-1, while the opposite was true in the 0% DSS control group (p < 0.05; Wilcoxon test). None of the treatment groups showed significant differences in alpha diversity between T-1 and T68 (Wilcoxon test). [file 40168_2020_933_MOESM4_ESM.pptx]

## Slide 1
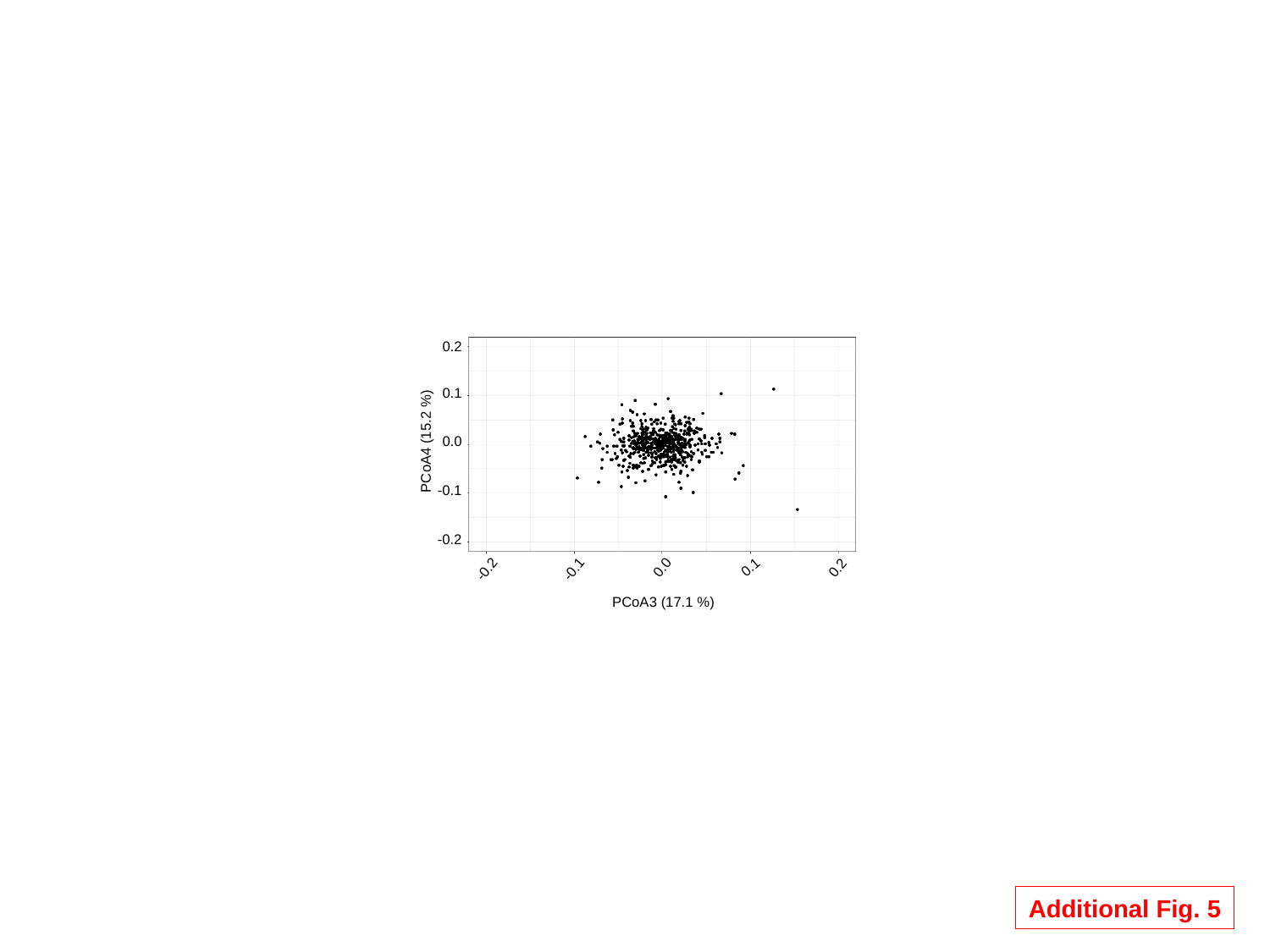

0.2
0.1
0.0
PCoA4 (15.2 %)
-0.1
-0.2
0.1
0.0
0.2
-0.1
-0.2
PCoA3 (17.1 %)
Additional Fig. 5

Supplement: Supplementary file 6 — Additional file 5 : Fig.5. Principal coordinates analysis. 3rd and 4th axes of the principal coordinates analysis of microbiota data shown in Fig. 2a. [file 40168_2020_933_MOESM5_ESM.pptx]

## Slide 1
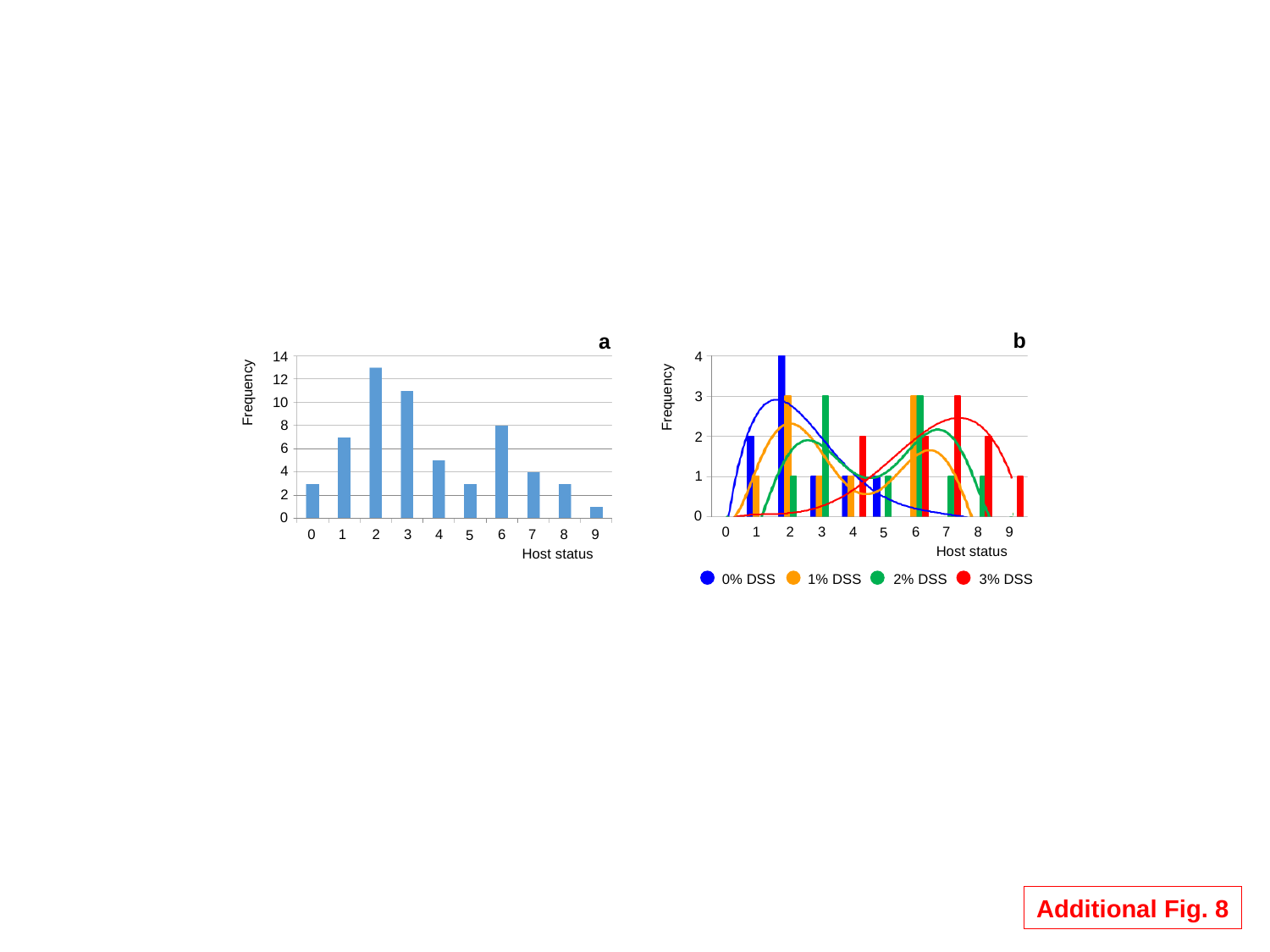

b
4
3
Frequency
2
1
0
9
8
0
1
2
3
4
6
7
5
Host status
0% DSS 1% DSS 2% DSS 3% DSS
a
14
12
Frequency
10
8
6
4
2
0
9
8
0
1
2
3
4
6
7
5
Host status
Additional Fig. 8

Supplement: Supplementary file 9 — Additional file 8 : Fig. 8. Two host states. Panel a, Host inflammatory status (distal colon histology score) distribution over all experimental groups (0% up to 3% DSS). Panel b, Distal colon histology score distributions for groups treated with 1, 2, or 3% DSS, and untreated control group. Colors represent treatment groups as indicated. Bimodal distributions are observed when data from all groups are combined (panel a, Additional Table 6), and within the 2% and 1% DSS groups (panel b). [file 40168_2020_933_MOESM8_ESM.pptx]
