# Supplementary material for: Alternative stable states in the intestinal ecosystem: proof of concept in a rat model and a perspective of therapeutic implications
Source: Microbiome. 2020 Nov 6;8:153. doi: 10.1186/s40168-020-00933-7 (PMC7646066; doi:10.1186/s40168-020-00933-7)
Supplement: Supplementary file 3 — Additional file 2 : Fig. 2. Effect of diet shift on microbiota composition. Panel a, Time course of relative abundance (number of sequence reads on a total of 38,000) of selected bacterial genera, before and after diet shift at T-31 (cf Additional Table 2). Colored lines each represent one rat. Dashed lines connect median values. Panel b, Abundance distributions of selected Lactobacillus species before (T-34) and after (T-20) diet shift (n=10). Abundance is expressed as number of sequence reads on a total of 38,000. Each dot represents one rat. P-values, Wilcoxon test. [file 40168_2020_933_MOESM2_ESM.pptx]

## Slide 1
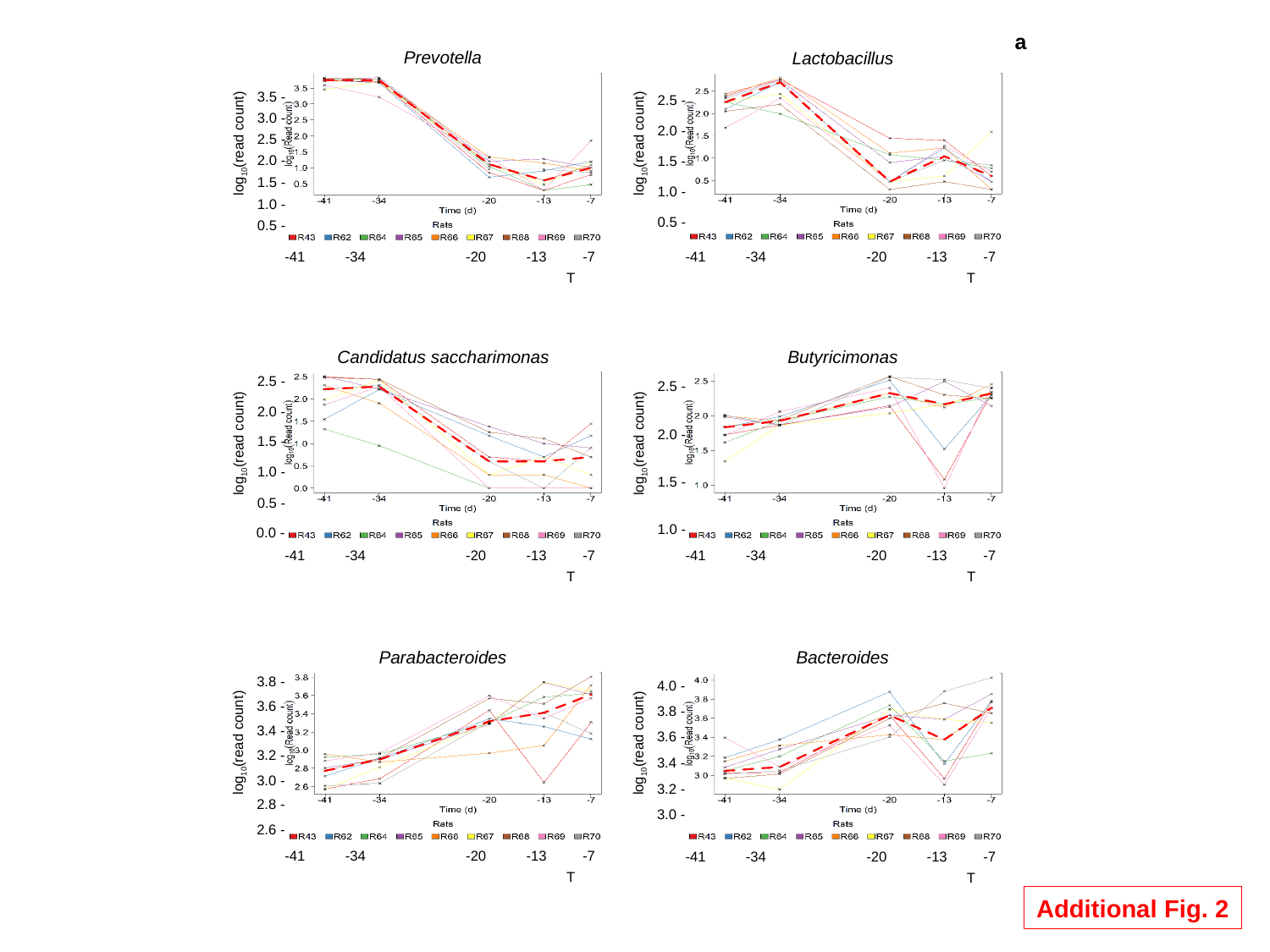

a
Prevotella
3.5 -
3.0 -
2.5 -
log10(read count)
2.0 -
1.5 -
1.0 -
0.5 -
-41 -34 -20 -13 -7
T
Lactobacillus
2.5 -
2.0 -
log10(read count)
1.5 -
1.0 -
0.5 -
-41 -34 -20 -13 -7
T
Butyricimonas
2.5 -
2.0 -
log10(read count)
1.5 -
1.0 -
-41 -34 -20 -13 -7
T
Candidatus saccharimonas
2.5 -
2.0 -
1.5 -
log10(read count)
1.0 -
0.5 -
0.0 -
-41 -34 -20 -13 -7
T
Parabacteroides
3.8 -
3.6 -
3.4 -
log10(read count)
3.2 -
3.0 -
2.8 -
2.6 -
-41 -34 -20 -13 -7
T
Bacteroides
4.0 -
3.8 -
3.6 -
log10(read count)
3.4 -
3.2 -
3.0 -
-41 -34 -20 -13 -7
T
Additional Fig. 2

## Slide 2
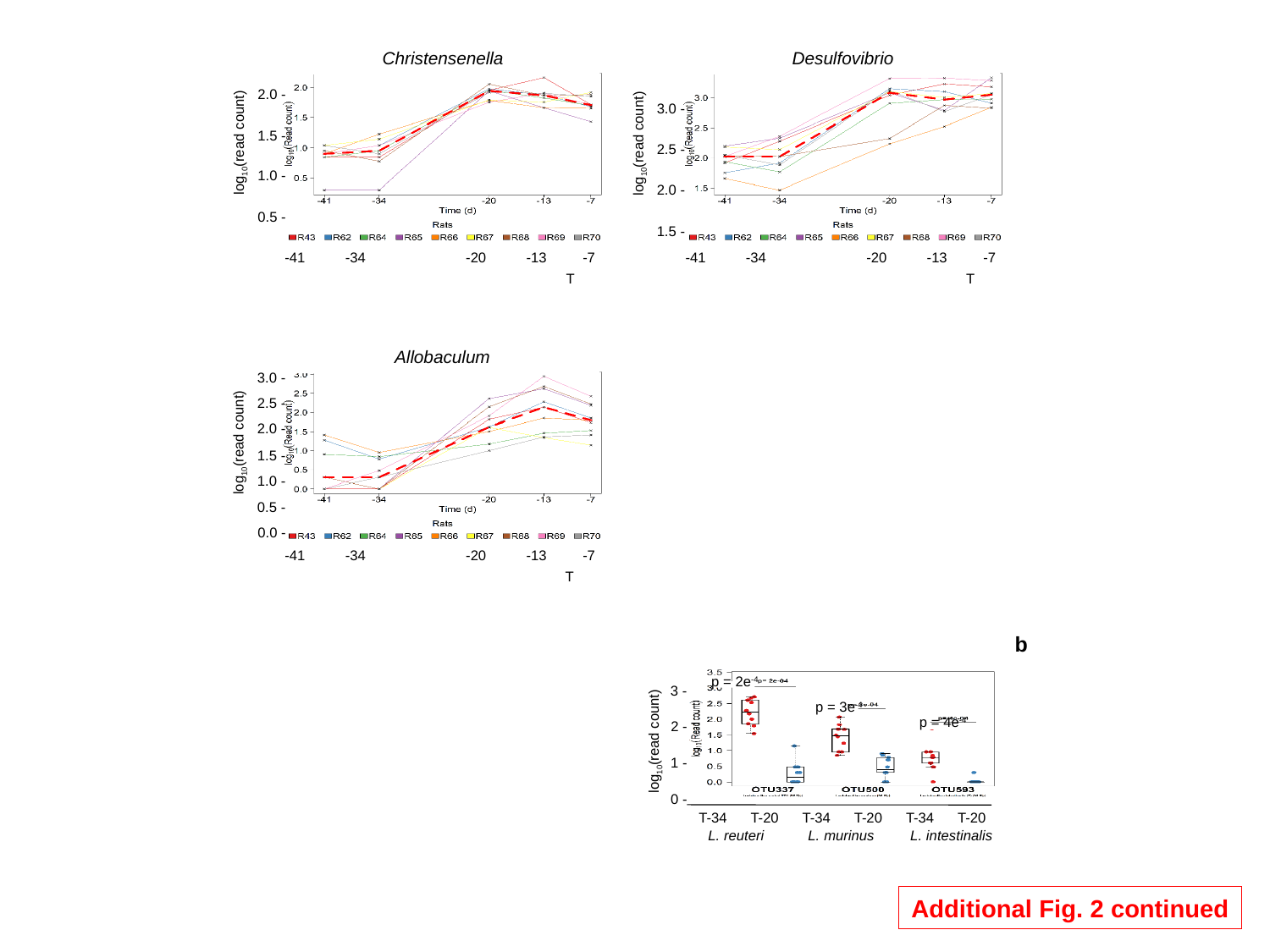

Desulfovibrio
3.0 -
log10(read count)
2.5 -
2.0 -
1.5 -
-41 -34 -20 -13 -7
T
Christensenella
2.0 -
1.5 -
log10(read count)
1.0 -
0.5 -
-41 -34 -20 -13 -7
T
Allobaculum
3.0 -
2.5 -
2.0 -
log10(read count)
1.5 -
1.0 -
0.5 -
0.0 -
-41 -34 -20 -13 -7
T
b
p = 2e-4
3 -
p = 3e-4
p = 4e-4
2 -
log10(read count)
1 -
0 -
T-34 T-20 T-34 T-20 T-34 T-20
L. reuteri L. murinus L. intestinalis
Additional Fig. 2 continued
