# Supplementary material for: Alternative stable states in the intestinal ecosystem: proof of concept in a rat model and a perspective of therapeutic implications
Source: Microbiome. 2020 Nov 6;8:153. doi: 10.1186/s40168-020-00933-7 (PMC7646066; doi:10.1186/s40168-020-00933-7)
Supplement: Supplementary file 7 — Additional file 6 : Fig. 6. Enterotyping. Microbiota data from T-7 to T68 were aggregated at genus-level, filtered for “unknown” and “uncultured” attributions, and analyzed using the clustering approach described in [6] (“enterotyping”). Panel a, clustering score (Calinski-Harabasz index [36]) as a function of the number of clusters shows a maximum at 3 clusters. Panel b, clustering with 3 clusters. Panel c, correspondence between clusters in panel b and PCoA1-based microbiota states from Fig. 2 (1, basal state; 2, alternative state). Numbers indicate number of samples (Table 1) in each category. Microbiota state 2 roughly corresponds to cluster A. Panel d, Akkermansia, Phascolarctobacterium and Bacteroides distributions in clusters A, B and C. Combined data from T-7 to T68; each dot represents one intestinal microbiota sample. Abundance is expressed as number of sequence reads on a total of 38,000. Only genera for which the median abundances in the two microbiota states differ at least 1.2-fold with q < 0.05 (Wilcoxon test with FDR adjustment) are presented. The table presents q-values for pairwise comparisons of relative abundances between clusters, for each of the three species (Kruskal-Wallis test with posthoc Dunn’s test and Holm correction for multiple comparisons). [file 40168_2020_933_MOESM6_ESM.pptx]

## Slide 1
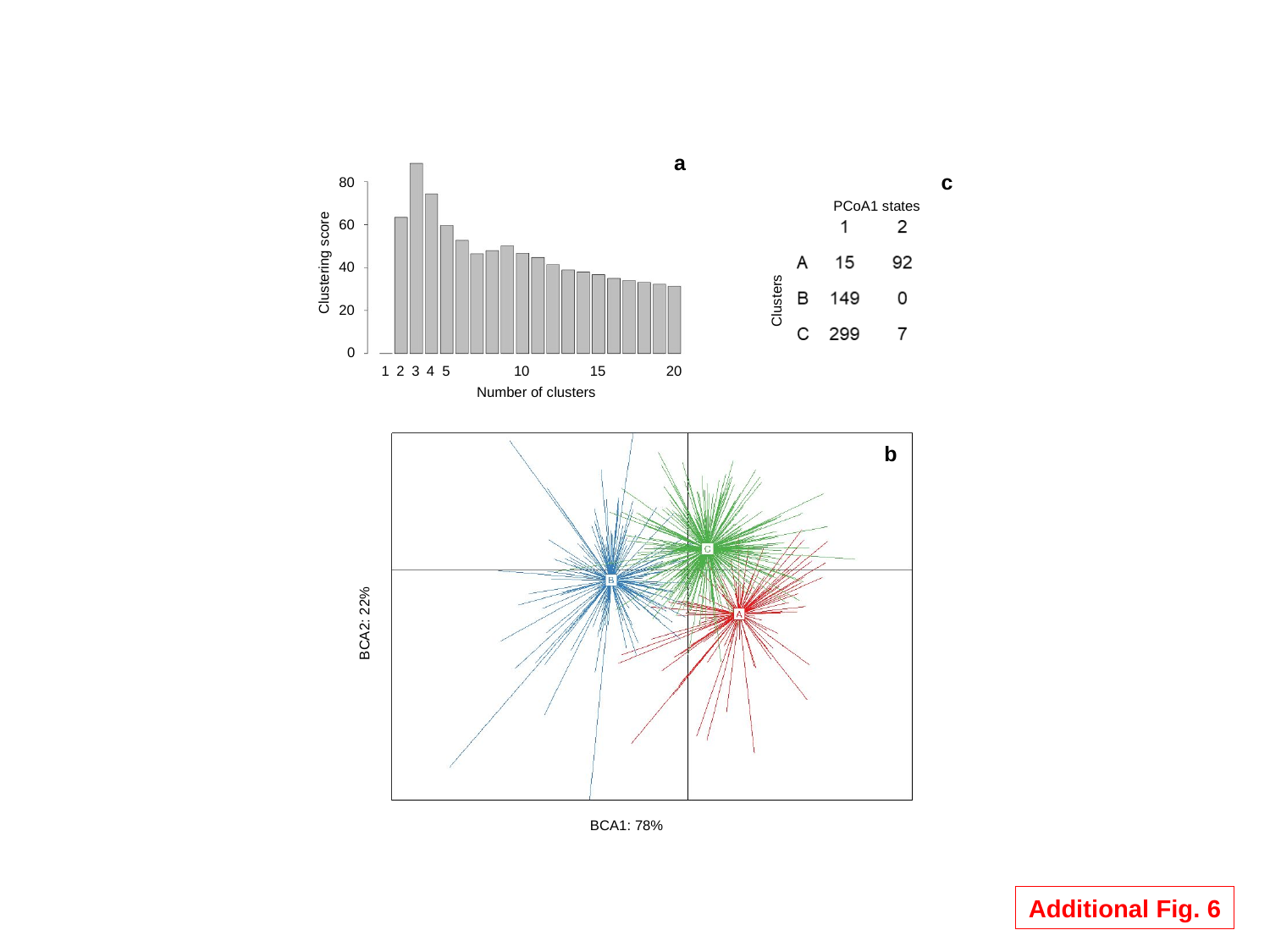

a
80
60
Clustering score
40
20
0
3
20
4
10
15
2
1
5
Number of clusters
c
PCoA1 states
Clusters
b
BCA2: 22%
BCA1: 78%
Additional Fig. 6

## Slide 2
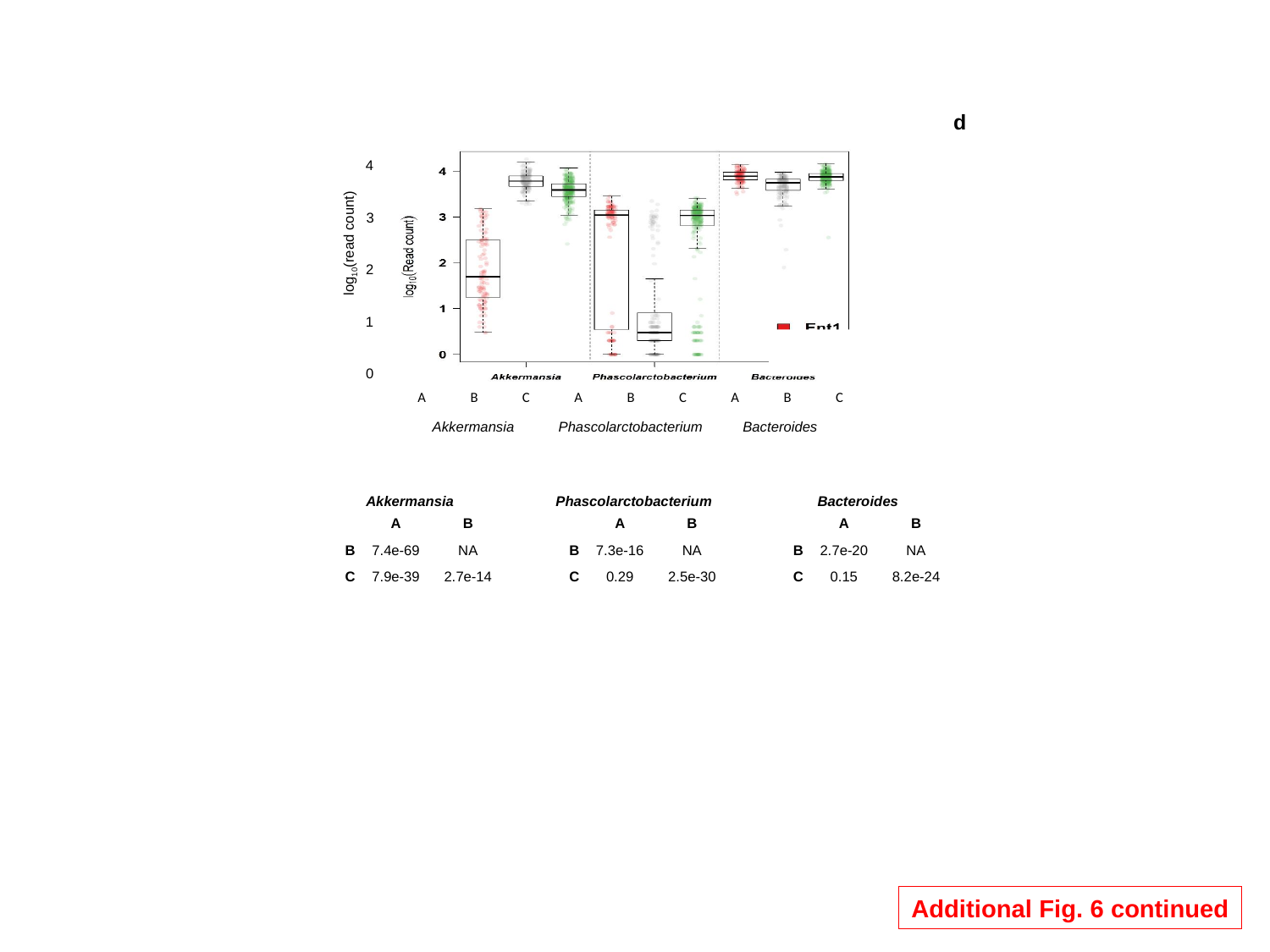

d
4
3
log10(read count)
2
1
0
Akkermansia Phascolarctobacterium Bacteroides
A B C A B C A B C
| Akkermansia | | | | Phascolarctobacterium | | | | Bacteroides | | |
| --- | --- | --- | --- | --- | --- | --- | --- | --- | --- | --- |
| | A | B | | | A | B | | | A | B |
| B | 7.4e-69 | NA | | B | 7.3e-16 | NA | | B | 2.7e-20 | NA |
| C | 7.9e-39 | 2.7e-14 | | C | 0.29 | 2.5e-30 | | C | 0.15 | 8.2e-24 |
Additional Fig. 6 continued
