# Supplementary material for: Alternative stable states in the intestinal ecosystem: proof of concept in a rat model and a perspective of therapeutic implications
Source: Microbiome. 2020 Nov 6;8:153. doi: 10.1186/s40168-020-00933-7 (PMC7646066; doi:10.1186/s40168-020-00933-7)
Supplement: Supplementary file 8 — Additional file 7 : Fig. 7. Correspondence between enterotyping-based clusters and PCoA1-based microbiota states. Juxtaposition of Table 1 (from main text, left) showing PCoA1-based microbiota states as a function of time for each rat, and the corresponding table showing enterotyping-based clusters from Additional Fig. 6b (right). Alternative microbiota state 2 and cluster A are highlighted. Rats (lines of the table) are grouped according to the host-ecosystem state at T68/75 (last column of Table 1, cf Fig. 5), then by resemblance of microbiota-by-time profile from T27 onward (cf Table 1). [file 40168_2020_933_MOESM7_ESM.pptx]

## Slide 1
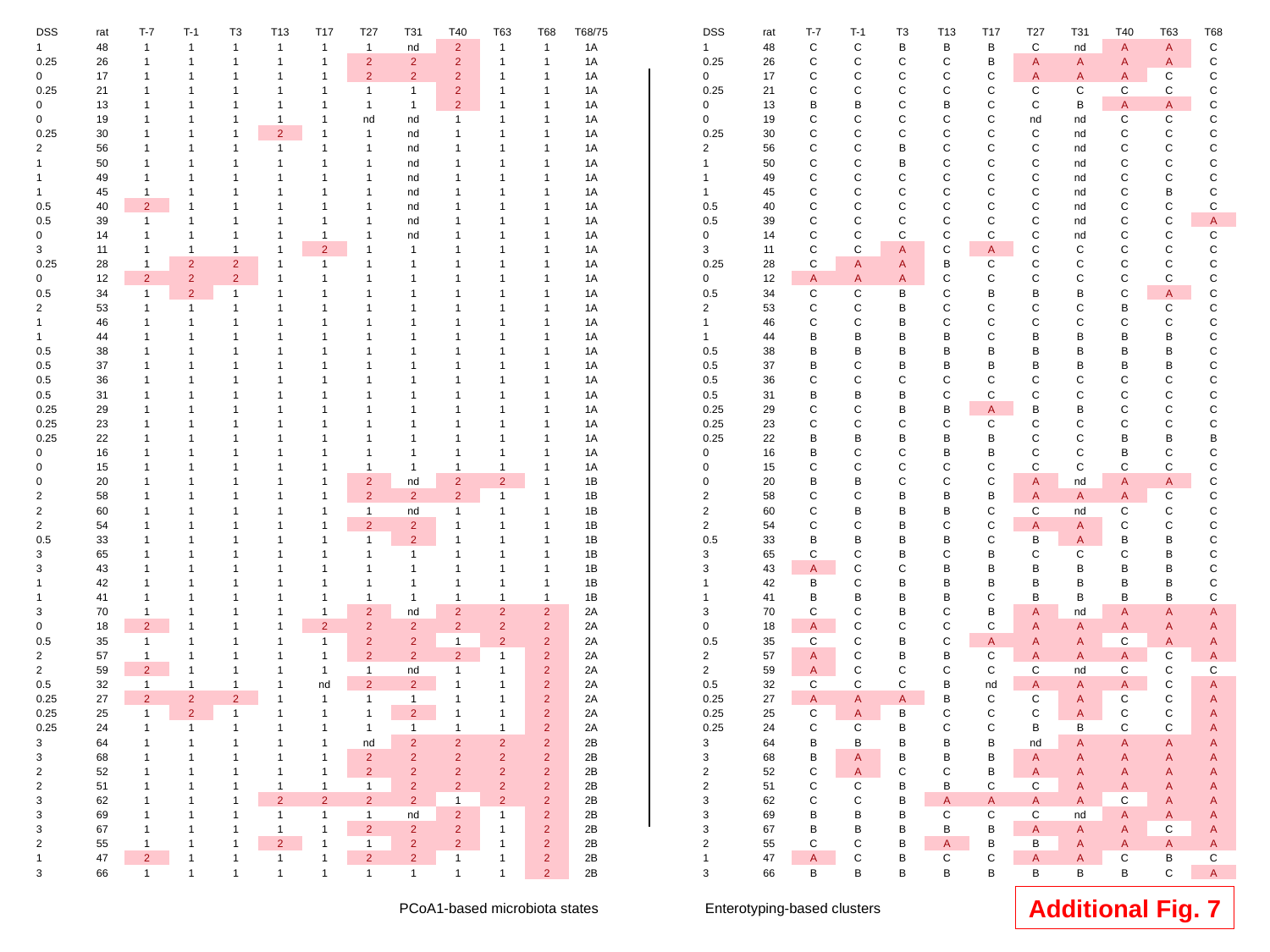

| DSS | rat | T-7 | T-1 | T3 | T13 | T17 | T27 | T31 | T40 | T63 | T68 | T68/75 | | | DSS | rat | T-7 | T-1 | T3 | T13 | T17 | T27 | T31 | T40 | T63 | T68 |
| --- | --- | --- | --- | --- | --- | --- | --- | --- | --- | --- | --- | --- | --- | --- | --- | --- | --- | --- | --- | --- | --- | --- | --- | --- | --- | --- |
| 1 | 48 | 1 | 1 | 1 | 1 | 1 | 1 | nd | 2 | 1 | 1 | 1A | | | 1 | 48 | C | C | B | B | B | C | nd | A | A | C |
| 0.25 | 26 | 1 | 1 | 1 | 1 | 1 | 2 | 2 | 2 | 1 | 1 | 1A | | | 0.25 | 26 | C | C | C | C | B | A | A | A | A | C |
| 0 | 17 | 1 | 1 | 1 | 1 | 1 | 2 | 2 | 2 | 1 | 1 | 1A | | | 0 | 17 | C | C | C | C | C | A | A | A | C | C |
| 0.25 | 21 | 1 | 1 | 1 | 1 | 1 | 1 | 1 | 2 | 1 | 1 | 1A | | | 0.25 | 21 | C | C | C | C | C | C | C | C | C | C |
| 0 | 13 | 1 | 1 | 1 | 1 | 1 | 1 | 1 | 2 | 1 | 1 | 1A | | | 0 | 13 | B | B | C | B | C | C | B | A | A | C |
| 0 | 19 | 1 | 1 | 1 | 1 | 1 | nd | nd | 1 | 1 | 1 | 1A | | | 0 | 19 | C | C | C | C | C | nd | nd | C | C | C |
| 0.25 | 30 | 1 | 1 | 1 | 2 | 1 | 1 | nd | 1 | 1 | 1 | 1A | | | 0.25 | 30 | C | C | C | C | C | C | nd | C | C | C |
| 2 | 56 | 1 | 1 | 1 | 1 | 1 | 1 | nd | 1 | 1 | 1 | 1A | | | 2 | 56 | C | C | B | C | C | C | nd | C | C | C |
| 1 | 50 | 1 | 1 | 1 | 1 | 1 | 1 | nd | 1 | 1 | 1 | 1A | | | 1 | 50 | C | C | B | C | C | C | nd | C | C | C |
| 1 | 49 | 1 | 1 | 1 | 1 | 1 | 1 | nd | 1 | 1 | 1 | 1A | | | 1 | 49 | C | C | C | C | C | C | nd | C | C | C |
| 1 | 45 | 1 | 1 | 1 | 1 | 1 | 1 | nd | 1 | 1 | 1 | 1A | | | 1 | 45 | C | C | C | C | C | C | nd | C | B | C |
| 0.5 | 40 | 2 | 1 | 1 | 1 | 1 | 1 | nd | 1 | 1 | 1 | 1A | | | 0.5 | 40 | C | C | C | C | C | C | nd | C | C | C |
| 0.5 | 39 | 1 | 1 | 1 | 1 | 1 | 1 | nd | 1 | 1 | 1 | 1A | | | 0.5 | 39 | C | C | C | C | C | C | nd | C | C | A |
| 0 | 14 | 1 | 1 | 1 | 1 | 1 | 1 | nd | 1 | 1 | 1 | 1A | | | 0 | 14 | C | C | C | C | C | C | nd | C | C | C |
| 3 | 11 | 1 | 1 | 1 | 1 | 2 | 1 | 1 | 1 | 1 | 1 | 1A | | | 3 | 11 | C | C | A | C | A | C | C | C | C | C |
| 0.25 | 28 | 1 | 2 | 2 | 1 | 1 | 1 | 1 | 1 | 1 | 1 | 1A | | | 0.25 | 28 | C | A | A | B | C | C | C | C | C | C |
| 0 | 12 | 2 | 2 | 2 | 1 | 1 | 1 | 1 | 1 | 1 | 1 | 1A | | | 0 | 12 | A | A | A | C | C | C | C | C | C | C |
| 0.5 | 34 | 1 | 2 | 1 | 1 | 1 | 1 | 1 | 1 | 1 | 1 | 1A | | | 0.5 | 34 | C | C | B | C | B | B | B | C | A | C |
| 2 | 53 | 1 | 1 | 1 | 1 | 1 | 1 | 1 | 1 | 1 | 1 | 1A | | | 2 | 53 | C | C | B | C | C | C | C | B | C | C |
| 1 | 46 | 1 | 1 | 1 | 1 | 1 | 1 | 1 | 1 | 1 | 1 | 1A | | | 1 | 46 | C | C | B | C | C | C | C | C | C | C |
| 1 | 44 | 1 | 1 | 1 | 1 | 1 | 1 | 1 | 1 | 1 | 1 | 1A | | | 1 | 44 | B | B | B | B | C | B | B | B | B | C |
| 0.5 | 38 | 1 | 1 | 1 | 1 | 1 | 1 | 1 | 1 | 1 | 1 | 1A | | | 0.5 | 38 | B | B | B | B | B | B | B | B | B | C |
| 0.5 | 37 | 1 | 1 | 1 | 1 | 1 | 1 | 1 | 1 | 1 | 1 | 1A | | | 0.5 | 37 | B | C | B | B | B | B | B | B | B | C |
| 0.5 | 36 | 1 | 1 | 1 | 1 | 1 | 1 | 1 | 1 | 1 | 1 | 1A | | | 0.5 | 36 | C | C | C | C | C | C | C | C | C | C |
| 0.5 | 31 | 1 | 1 | 1 | 1 | 1 | 1 | 1 | 1 | 1 | 1 | 1A | | | 0.5 | 31 | B | B | B | C | C | C | C | C | C | C |
| 0.25 | 29 | 1 | 1 | 1 | 1 | 1 | 1 | 1 | 1 | 1 | 1 | 1A | | | 0.25 | 29 | C | C | B | B | A | B | B | C | C | C |
| 0.25 | 23 | 1 | 1 | 1 | 1 | 1 | 1 | 1 | 1 | 1 | 1 | 1A | | | 0.25 | 23 | C | C | C | C | C | C | C | C | C | C |
| 0.25 | 22 | 1 | 1 | 1 | 1 | 1 | 1 | 1 | 1 | 1 | 1 | 1A | | | 0.25 | 22 | B | B | B | B | B | C | C | B | B | B |
| 0 | 16 | 1 | 1 | 1 | 1 | 1 | 1 | 1 | 1 | 1 | 1 | 1A | | | 0 | 16 | B | C | C | B | B | C | C | B | C | C |
| 0 | 15 | 1 | 1 | 1 | 1 | 1 | 1 | 1 | 1 | 1 | 1 | 1A | | | 0 | 15 | C | C | C | C | C | C | C | C | C | C |
| 0 | 20 | 1 | 1 | 1 | 1 | 1 | 2 | nd | 2 | 2 | 1 | 1B | | | 0 | 20 | B | B | C | C | C | A | nd | A | A | C |
| 2 | 58 | 1 | 1 | 1 | 1 | 1 | 2 | 2 | 2 | 1 | 1 | 1B | | | 2 | 58 | C | C | B | B | B | A | A | A | C | C |
| 2 | 60 | 1 | 1 | 1 | 1 | 1 | 1 | nd | 1 | 1 | 1 | 1B | | | 2 | 60 | C | B | B | B | C | C | nd | C | C | C |
| 2 | 54 | 1 | 1 | 1 | 1 | 1 | 2 | 2 | 1 | 1 | 1 | 1B | | | 2 | 54 | C | C | B | C | C | A | A | C | C | C |
| 0.5 | 33 | 1 | 1 | 1 | 1 | 1 | 1 | 2 | 1 | 1 | 1 | 1B | | | 0.5 | 33 | B | B | B | B | C | B | A | B | B | C |
| 3 | 65 | 1 | 1 | 1 | 1 | 1 | 1 | 1 | 1 | 1 | 1 | 1B | | | 3 | 65 | C | C | B | C | B | C | C | C | B | C |
| 3 | 43 | 1 | 1 | 1 | 1 | 1 | 1 | 1 | 1 | 1 | 1 | 1B | | | 3 | 43 | A | C | C | B | B | B | B | B | B | C |
| 1 | 42 | 1 | 1 | 1 | 1 | 1 | 1 | 1 | 1 | 1 | 1 | 1B | | | 1 | 42 | B | C | B | B | B | B | B | B | B | C |
| 1 | 41 | 1 | 1 | 1 | 1 | 1 | 1 | 1 | 1 | 1 | 1 | 1B | | | 1 | 41 | B | B | B | B | C | B | B | B | B | C |
| 3 | 70 | 1 | 1 | 1 | 1 | 1 | 2 | nd | 2 | 2 | 2 | 2A | | | 3 | 70 | C | C | B | C | B | A | nd | A | A | A |
| 0 | 18 | 2 | 1 | 1 | 1 | 2 | 2 | 2 | 2 | 2 | 2 | 2A | | | 0 | 18 | A | C | C | C | C | A | A | A | A | A |
| 0.5 | 35 | 1 | 1 | 1 | 1 | 1 | 2 | 2 | 1 | 2 | 2 | 2A | | | 0.5 | 35 | C | C | B | C | A | A | A | C | A | A |
| 2 | 57 | 1 | 1 | 1 | 1 | 1 | 2 | 2 | 2 | 1 | 2 | 2A | | | 2 | 57 | A | C | B | B | C | A | A | A | C | A |
| 2 | 59 | 2 | 1 | 1 | 1 | 1 | 1 | nd | 1 | 1 | 2 | 2A | | | 2 | 59 | A | C | C | C | C | C | nd | C | C | C |
| 0.5 | 32 | 1 | 1 | 1 | 1 | nd | 2 | 2 | 1 | 1 | 2 | 2A | | | 0.5 | 32 | C | C | C | B | nd | A | A | A | C | A |
| 0.25 | 27 | 2 | 2 | 2 | 1 | 1 | 1 | 1 | 1 | 1 | 2 | 2A | | | 0.25 | 27 | A | A | A | B | C | C | A | C | C | A |
| 0.25 | 25 | 1 | 2 | 1 | 1 | 1 | 1 | 2 | 1 | 1 | 2 | 2A | | | 0.25 | 25 | C | A | B | C | C | C | A | C | C | A |
| 0.25 | 24 | 1 | 1 | 1 | 1 | 1 | 1 | 1 | 1 | 1 | 2 | 2A | | | 0.25 | 24 | C | C | B | C | C | B | B | C | C | A |
| 3 | 64 | 1 | 1 | 1 | 1 | 1 | nd | 2 | 2 | 2 | 2 | 2B | | | 3 | 64 | B | B | B | B | B | nd | A | A | A | A |
| 3 | 68 | 1 | 1 | 1 | 1 | 1 | 2 | 2 | 2 | 2 | 2 | 2B | | | 3 | 68 | B | A | B | B | B | A | A | A | A | A |
| 2 | 52 | 1 | 1 | 1 | 1 | 1 | 2 | 2 | 2 | 2 | 2 | 2B | | | 2 | 52 | C | A | C | C | B | A | A | A | A | A |
| 2 | 51 | 1 | 1 | 1 | 1 | 1 | 1 | 2 | 2 | 2 | 2 | 2B | | | 2 | 51 | C | C | B | B | C | C | A | A | A | A |
| 3 | 62 | 1 | 1 | 1 | 2 | 2 | 2 | 2 | 1 | 2 | 2 | 2B | | | 3 | 62 | C | C | B | A | A | A | A | C | A | A |
| 3 | 69 | 1 | 1 | 1 | 1 | 1 | 1 | nd | 2 | 1 | 2 | 2B | | | 3 | 69 | B | B | B | C | C | C | nd | A | A | A |
| 3 | 67 | 1 | 1 | 1 | 1 | 1 | 2 | 2 | 2 | 1 | 2 | 2B | | | 3 | 67 | B | B | B | B | B | A | A | A | C | A |
| 2 | 55 | 1 | 1 | 1 | 2 | 1 | 1 | 2 | 2 | 1 | 2 | 2B | | | 2 | 55 | C | C | B | A | B | B | A | A | A | A |
| 1 | 47 | 2 | 1 | 1 | 1 | 1 | 2 | 2 | 1 | 1 | 2 | 2B | | | 1 | 47 | A | C | B | C | C | A | A | C | B | C |
| 3 | 66 | 1 | 1 | 1 | 1 | 1 | 1 | 1 | 1 | 1 | 2 | 2B | | | 3 | 66 | B | B | B | B | B | B | B | B | C | A |
PCoA1-based microbiota states
Enterotyping-based clusters
Additional Fig. 7
