# Supplementary material for: Alternative stable states in the intestinal ecosystem: proof of concept in a rat model and a perspective of therapeutic implications
Source: Microbiome. 2020 Nov 6;8:153. doi: 10.1186/s40168-020-00933-7 (PMC7646066; doi:10.1186/s40168-020-00933-7)
Supplement: Supplementary file 10 — Additional file 9 : Fig. 9. State transitions in the host-microbiota ecosystem. Host-microbiota ecosystem model from Fig. 5. Possible trajectories of the host-microbiota ecosystem from a “healthy” state to a “(pre-) disease” state, comprising a microbiota state-transition and a host inflammatory state-transition, are indicated by the three filled arrows. These transitions may be sequential (upper and lower arrows) or simultaneous (diagonal arrow). [file 40168_2020_933_MOESM9_ESM.pptx]

## Slide 1
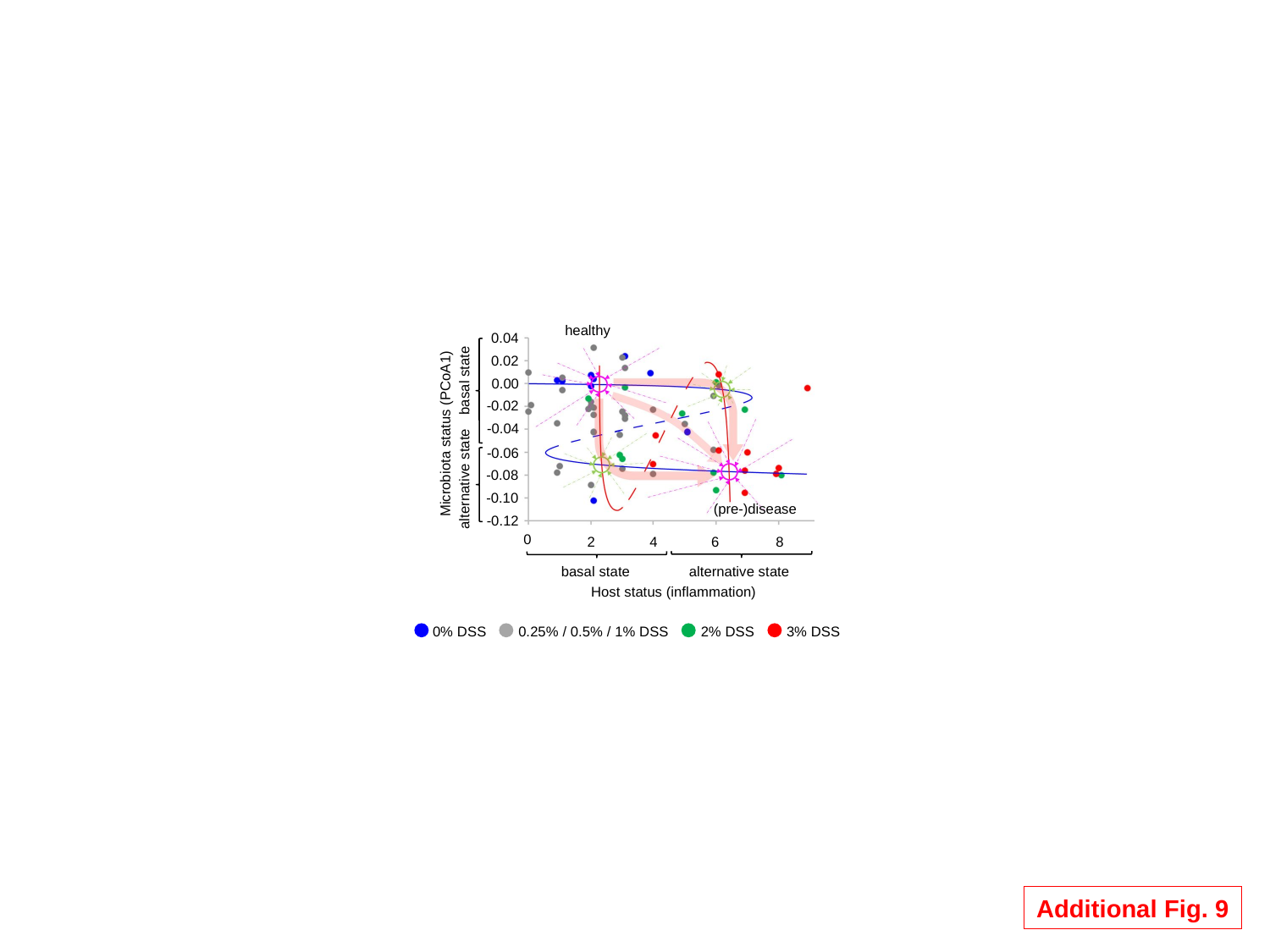

healthy
0.04
0.02
0.00
-0.02
-0.04
-0.06
-0.08
-0.10
-0.12
0
2
4
8
6
(pre-)disease
0% DSS 0.25% / 0.5% / 1% DSS 2% DSS 3% DSS
basal state
Microbiota status (PCoA1)
alternative state
basal state
alternative state
Host status (inflammation)
Additional Fig. 9
