# Supplementary material for: Alternative stable states in the intestinal ecosystem: proof of concept in a rat model and a perspective of therapeutic implications
Source: Microbiome. 2020 Nov 6;8:153. doi: 10.1186/s40168-020-00933-7 (PMC7646066; doi:10.1186/s40168-020-00933-7)
Supplement: Supplementary file 11 — Additional file 10 : Fig. 10. Constitution of experimental groups. Panel a, Composition of experimental groups regarding microbiota composition at T-49 (Bacteroides+Prevotella group as percentage of total bacteria, determined by qPCR), cage occupancy at the provider, and affiliation (litter). % DSS indicates experimental groups (DSS treatments between T0 and T30). Panel b, Correlation between Bacteroides+Prevotella content determined by qPCR and determined by MiSeq on the same DNA samples (Spearman's rank correlation: Rho = 0.77, p < 0.05). Panel c, Composition of experimental groups regarding body weight at T-31. No significant differences were observed between groups (ANOVA, p = 0.7). [file 40168_2020_933_MOESM10_ESM.pptx]

## Slide 1
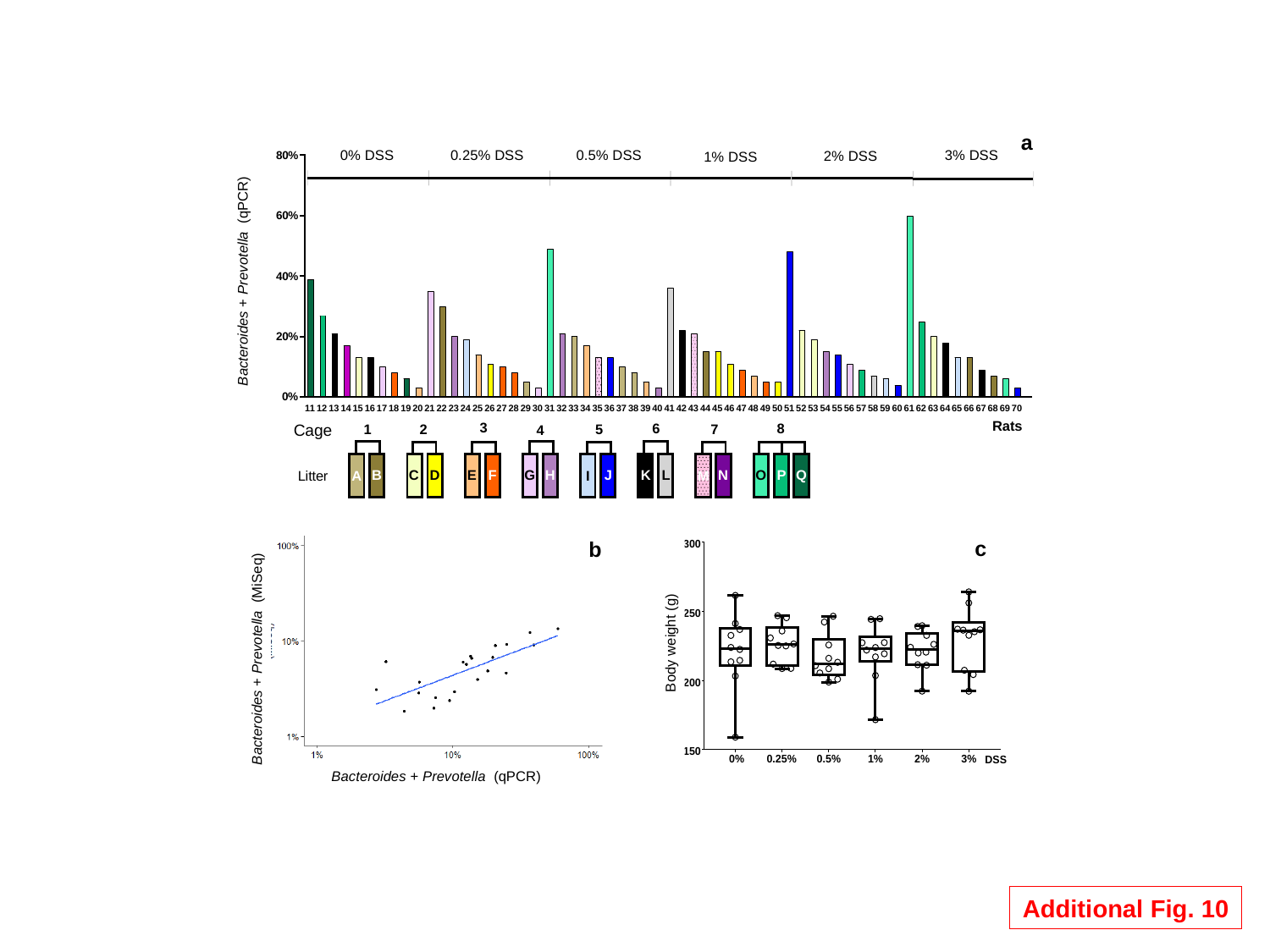

a
0% DSS
0.25% DSS
0.5% DSS
3% DSS
2% DSS
1% DSS
Bacteroides + Prevotella (qPCR)
Bacteroides + Prevotella (MiSeq)
Bacteroides + Prevotella (qPCR)
Body weight (g)
c
b
Additional Fig. 10
