# Supplementary material for: Alternative stable states in the intestinal ecosystem: proof of concept in a rat model and a perspective of therapeutic implications
Source: Microbiome. 2020 Nov 6;8:153. doi: 10.1186/s40168-020-00933-7 (PMC7646066; doi:10.1186/s40168-020-00933-7)
Supplement: Supplementary file 12 — Additional file 11 : Table 1. Diet composition. Description of diets used. [file 40168_2020_933_MOESM11_ESM.docx]

| **Additional Table 1. Diet composition.** |  |  |
| --- | --- | --- |
|  |  |  |
|  | *Diet 1 | **Diet 2 |
|  | % | % |
| Crude protein | 22.0 | 17.6 |
| Crude fat | 4.5 | 7.1 |
| Crude fiber (cellulose, hemicelluloses, lignin) | 3.9 | 0 |
| Crude ash | 6.7 | 3.2 |
| N free extracts (carbohydrates) | 50.0 | 68.2 |
| Starch | 34.9 | 38.5 |
| Sugar | 5.2 | 13.1 |
| Dextrin | nd | 15.8 |
|  |  |  |
|  |  |  |
| Metabolizable Energy (ME - Atwater; MJ/kg) | 14.0 | 17.1 |
| Protein (%) | 26 | 17 |
| Fat (%) | 12 | 16 |
| Carbohydrates (%) | 62 | 67 |

* M-Z diet (ssniff, Soest, Germany), **AIN93G diet w/o cellulose (ssniff). Nd, not determined.
