# Supplementary material for: Alternative stable states in the intestinal ecosystem: proof of concept in a rat model and a perspective of therapeutic implications
Source: Microbiome. 2020 Nov 6;8:153. doi: 10.1186/s40168-020-00933-7 (PMC7646066; doi:10.1186/s40168-020-00933-7)
Supplement: Supplementary file 13 — Additional file 12 : Table 2. Effect of diet shift on microbiota composition. Differential median relative abundances before (T-34) and after (T-20) diet shift for selected bacterial genera. [file 40168_2020_933_MOESM12_ESM.docx]

**Additional Table 2. Effect of diet shift on microbiota composition.**

|  | median T-34 | median T-20 | T-20 / T-34 | q |
| --- | --- | --- | --- | --- |
| *Prevotella* | 5345 | 12 | 0.002 | 0.014 |
| *Lactobacillus* | 384 | 4 | 0.010 | 0.013 |
| *Candidatus saccharimonas* | 186 | 2 | 0.010 | 0.033 |
| *Butyricimonas* | 83 | 218 | 2.6 | 0.025 |
| *Parabacteroides* | 801 | 2135 | 2.6 | 0.026 |
| *Bacteroides* | 1404 | 4280 | 3.0 | 0.034 |
| *Christensenella* | 9 | 88 | 9.7 | 0.014 |
| *Desulfovibrio* | 100 | 1241 | 12.4 | 0.060 |
| *Allobaculum* | 2 | 53 | 35.3 | 0.013 |

Differential median abundances before (T-34) and after (T-20) diet shift for selected bacterial genera. Only genera for which the median abundances (number of sequence reads on a total of 38,000) differ with q < 0.1 (Wilcoxon test with FDR adjustment) are presented. Genera for which both median abundances are smaller than 10 are omitted. See also Suppl. Fig. 2a for time course of abundances, with distribution of observations at each time-point.
