# Supplementary material for: Alternative stable states in the intestinal ecosystem: proof of concept in a rat model and a perspective of therapeutic implications
Source: Microbiome. 2020 Nov 6;8:153. doi: 10.1186/s40168-020-00933-7 (PMC7646066; doi:10.1186/s40168-020-00933-7)
Supplement: Supplementary file 14 — Additional file 13 : Table 3. Bimodal distribution of microbiota status. Statistical support for bimodal distribution of microbiota status (implying two microbiota states). [file 40168_2020_933_MOESM13_ESM.docx]

**Additional Table 3. Bimodal distribution of microbiota status.**

| Classes considered | BIC | ICL |
| --- | --- | --- |
| 1 | 856.5 | 856.5 |
| 2 | 885.6 | 876.1 |
| 3 | 870.9 | 767.6 |

The distribution of microbiota status in data from all experimental groups (0% to 3% DSS) for time-points T-7, T-1, T63 and T68 (Fig. 2c, top left graph) was analyzed using Mclust5.4 [18]. Maximal values of the information criteria BIC (Bayesian Information Criterion) and ICL (integrated complete-data likelihood criterion) both indicate the same model (2 classes, i.e., a bimodal distribution) as the best fit. Likelihood ratio testing (LRT) using bootstrap sequential LRT as implemented in Mclust5.4 [18] (model “V”, 999 replications) confirms a bimodal distribution as the best fit (p=0.001).
