# Supplementary material for: Alternative stable states in the intestinal ecosystem: proof of concept in a rat model and a perspective of therapeutic implications
Source: Microbiome. 2020 Nov 6;8:153. doi: 10.1186/s40168-020-00933-7 (PMC7646066; doi:10.1186/s40168-020-00933-7)
Supplement: Supplementary file 15 — Additional file 14 : Table 4. Distal colon histology scoring criteria. Scoring criteria for evaluation of inflammatory status. [file 40168_2020_933_MOESM14_ESM.docx]

**Additional Table 4. Distal colon histology scoring criteria.**

| Edema | 0/1/2 |
| --- | --- |
| Hyperplasia of mucosa | 0/1/2 |
| Epithelial atrophy | 0/1/2 |
| Ulceration | 0/1/2 |
| Cryptic abscess | 0/1/2/3/4/5 |
| Intestinal wall thickness | 0/1/2/3/4/5 |
| Squamous metaplasia | 0/1 |
| Polyps | 0/1 |
| Mononuclear cell infiltration | 0/1/2/3/4/5 |
| Neutrophils infiltration | 0/1/2/3/4/5 |
| Total | from 0 to 30 |

Host inflammatory status was evaluated by examination of distal colon tissue, attributing one of the indicated scores for each of the criteria listed. Addition of the scores for individual criteria gives a total score with a theoretical maximum of 30.
