# Supplementary material for: Alternative stable states in the intestinal ecosystem: proof of concept in a rat model and a perspective of therapeutic implications
Source: Microbiome. 2020 Nov 6;8:153. doi: 10.1186/s40168-020-00933-7 (PMC7646066; doi:10.1186/s40168-020-00933-7)
Supplement: Supplementary file 16 — Additional file 15 : Table 5. Distal colon histology scores. [file 40168_2020_933_MOESM15_ESM.docx]

**Additional Table 5. Distal colon histology scores.**

| **% DSS** | **Rat nr** | **Edema** | **Hyperplasia of mucosa** | **Epithelial atrophy** | **Ulceration** | **Cryptic abscess** | **Intestinal wall thickness** | **Squamous metaplasia** | **Polyps** | **Mononuclear cell**  **infiltration** | **Neutrophils infiltration** | **Total score** |
| --- | --- | --- | --- | --- | --- | --- | --- | --- | --- | --- | --- | --- |
|  |  |  |  |  |  |  |  |  |  |  |  |  |
| 0 | 12 | 0 | 0 | 1 | 0 | 0 | 0 | 0 | 0 | 1 | 0 | 2 |
| 0 | 13 | 0 | 0 | 0 | 0 | 0 | 0 | 0 | 0 | 2 | 0 | 2 |
| 0 | 14 | 0 | 0 | 1 | 0 | 0 | 0 | 0 | 0 | 0 | 0 | 1 |
| 0 | 15 | 0 | 0 | 0 | 0 | 0 | 1 | 0 | 0 | 1 | 1 | 3 |
| 0 | 16 | 0 | 0 | 0 | 0 | 0 | 0 | 0 | 0 | 0 | 1 | 1 |
| 0 | 17 | 0 | 0 | 1 | 0 | 0 | 0 | 0 | 0 | 2 | 1 | 4 |
| 0 | 18 | 0 | 0 | 0 | 0 | 0 | 0 | 0 | 0 | 1 | 1 | 2 |
| 0 | 19 | 0 | 0 | 1 | 0 | 0 | 0 | 0 | 0 | 1 | 0 | 2 |
| 0 | 20 | 0 | 0 | 2 | 0 | 0 | 0 | 0 | 0 | 2 | 1 | 5 |
| 0.25 | 21 | 0 | 0 | 1 | 0 | 0 | 0 | 0 | 0 | 2 | 0 | 3 |
| 0.25 | 22 | 0 | 0 | 0 | 0 | 0 | 0 | 0 | 0 | 1 | 0 | 1 |
| 0.25 | 23 | 0 | 0 | 0 | 0 | 0 | 0 | 0 | 0 | 0 | 0 | 0 |
| 0.25 | 24 | 0 | 0 | 0 | 0 | 0 | 1 | 0 | 0 | 1 | 0 | 2 |
| 0.25 | 25 | 0 | 0 | 2 | 0 | 0 | 0 | 0 | 0 | 2 | 0 | 4 |
| 0.25 | 26 | 0 | 0 | 1 | 0 | 0 | 0 | 0 | 0 | 2 | 0 | 3 |
| 0.25 | 27 | 0 | 0 | 1 | 0 | 0 | 0 | 0 | 0 | 2 | 0 | 3 |
| 0.25 | 28 | 0 | 0 | 1 | 0 | 0 | 0 | 0 | 0 | 1 | 0 | 2 |
| 0.25 | 29 | 0 | 0 | 1 | 0 | 0 | 0 | 0 | 0 | 2 | 0 | 3 |
| 0.25 | 30 | 0 | 0 | 1 | 0 | 0 | 0 | 0 | 0 | 2 | 0 | 3 |
| 0.5 | 31 | 0 | 0 | 1 | 0 | 0 | 0 | 0 | 0 | 1 | 0 | 2 |
| 0.5 | 32 | 0 | 0 | 1 | 0 | 0 | 0 | 0 | 0 | 0 | 0 | 1 |
| 0.5 | 33 | 0 | 0 | 2 | 0 | 0 | 0 | 0 | 0 | 2 | 1 | 5 |
| 0.5 | 34 | 1 | 0 | 0 | 0 | 0 | 1 | 0 | 0 | 1 | 0 | 3 |
| 0.5 | 35 | 0 | 0 | 0 | 0 | 0 | 0 | 0 | 0 | 1 | 0 | 1 |
| 0.5 | 36 | 0 | 0 | 0 | 0 | 0 | 0 | 0 | 0 | 0 | 0 | 0 |
| 0.5 | 37 | 0 | 0 | 1 | 0 | 0 | 0 | 0 | 0 | 0 | 0 | 1 |
| 0.5 | 38 | 0 | 0 | 1 | 0 | 0 | 0 | 0 | 0 | 1 | 0 | 2 |
| 0.5 | 39 | 0 | 0 | 1 | 0 | 0 | 0 | 0 | 0 | 1 | 0 | 2 |
| 0.5 | 40 | 0 | 0 | 0 | 0 | 0 | 0 | 0 | 0 | 0 | 0 | 0 |
| 1 | 41 | 1 | 0 | 2 | 0 | 0 | 0 | 0 | 0 | 3 | 0 | 6 |
| 1 | 42 | 1 | 0 | 2 | 0 | 0 | 0 | 0 | 0 | 3 | 0 | 6 |
| 1 | 44 | 0 | 0 | 2 | 0 | 0 | 0 | 0 | 0 | 2 | 0 | 4 |
| 1 | 45 | 0 | 0 | 1 | 0 | 0 | 0 | 0 | 0 | 0 | 0 | 1 |
| 1 | 46 | 0 | 0 | 1 | 0 | 0 | 0 | 0 | 0 | 1 | 0 | 2 |
| 1 | 47 | 1 | 0 | 2 | 1 | 0 | 0 | 0 | 0 | 2 | 0 | 6 |
| 1 | 48 | 0 | 0 | 1 | 0 | 0 | 0 | 0 | 0 | 2 | 0 | 3 |
| 1 | 49 | 0 | 0 | 1 | 0 | 0 | 0 | 0 | 0 | 1 | 0 | 2 |
| 1 | 50 | 0 | 0 | 0 | 0 | 0 | 0 | 0 | 0 | 2 | 0 | 2 |
| 2 | 51 | 1 | 0 | 2 | 0 | 0 | 0 | 0 | 0 | 3 | 2 | 8 |
| 2 | 52 | 1 | 0 | 2 | 0 | 0 | 0 | 0 | 0 | 3 | 0 | 6 |
| 2 | 53 | 0 | 0 | 1 | 0 | 0 | 0 | 0 | 0 | 2 | 0 | 3 |
| 2 | 54 | 1 | 0 | 2 | 1 | 0 | 0 | 0 | 0 | 3 | 0 | 7 |
| 2 | 55 | 1 | 0 | 2 | 0 | 0 | 0 | 0 | 0 | 3 | 0 | 6 |
| 2 | 56 | 0 | 0 | 1 | 0 | 0 | 0 | 0 | 0 | 1 | 0 | 2 |
| 2 | 57 | 0 | 0 | 1 | 0 | 0 | 0 | 0 | 0 | 2 | 0 | 3 |
| 2 | 58 | 0 | 0 | 2 | 0 | 0 | 0 | 0 | 0 | 2 | 1 | 5 |
| 2 | 59 | 0 | 0 | 1 | 0 | 0 | 0 | 0 | 0 | 2 | 0 | 3 |
| 2 | 60 | 1 | 0 | 1 | 1 | 0 | 0 | 0 | 0 | 3 | 0 | 6 |
| 3 | 11 | 1 | 0 | 2 | 0 | 0 | 0 | 0 | 0 | 1 | 0 | 4 |
| 3 | 43 | 1 | 0 | 2 | 1 | 0 | 0 | 0 | 0 | 2 | 0 | 6 |
| 3 | 62 | 1 | 0 | 2 | 1 | 0 | 0 | 0 | 0 | 3 | 0 | 7 |
| 3 | 64 | 1 | 0 | 2 | 1 | 0 | 0 | 0 | 0 | 3 | 0 | 7 |
| 3 | 65 | 1 | 0 | 2 | 1 | 0 | 0 | 0 | 0 | 4 | 1 | 9 |
| 3 | 66 | 1 | 0 | 2 | 1 | 0 | 0 | 0 | 0 | 3 | 1 | 8 |
| 3 | 67 | 1 | 0 | 2 | 1 | 0 | 0 | 0 | 0 | 3 | 0 | 7 |
| 3 | 68 | 2 | 0 | 2 | 1 | 0 | 0 | 0 | 0 | 3 | 0 | 8 |
| 3 | 69 | 1 | 0 | 2 | 0 | 0 | 0 | 0 | 0 | 3 | 0 | 6 |
| 3 | 70 | 0 | 0 | 2 | 0 | 0 | 0 | 0 | 0 | 2 | 0 | 4 |

Histology scores attributed to individual rats (distal colon) for each of the criteria listed. % DSS indicates the treatment group.
