# Supplementary material for: Alternative stable states in the intestinal ecosystem: proof of concept in a rat model and a perspective of therapeutic implications
Source: Microbiome. 2020 Nov 6;8:153. doi: 10.1186/s40168-020-00933-7 (PMC7646066; doi:10.1186/s40168-020-00933-7)
Supplement: Supplementary file 17 — Additional file 16 : Table 6. Bimodal distribution of host inflammatory status. Statistical support for bimodal distribution of host inflammatory status (implying two host states). [file 40168_2020_933_MOESM16_ESM.docx]

**Additional Table 6. Bimodal distribution of host inflammatory status.**

| Classes considered | BIC | ICL |
| --- | --- | --- |
| 1 | -269.9 | -269.9 |
| 2 | -263.1 | -266.4 |
| 3 | -271.2 | -293.6 |

The distribution of host inflammatory status in data from all experimental groups (0% to 3% DSS) at T75 (Fig. 4a) was analyzed using Mclust5.4 [18]. Maximal (here: least negative) values of the information criteria BIC (Bayesian Information Criterion) and ICL (integrated complete-data likelihood criterion) both indicate the same model (2 classes, i.e., a bimodal distribution) as the best fit. Likelihood ratio testing (LRT) using bootstrap sequential LRT as implemented in Mclust5.4 [18] (model “E”, 999 replications) confirms a bimodal distribution as the best fit (p=0.001).
